# Hadal Snailfishes (Teleostei: Liparidae) Extend Across Multiple Trenches: Molecular Insights and Implications for Taxonomic Nomenclature

**DOI:** 10.1002/ece3.71779

**Published:** 2025-09-29

**Authors:** Paige J. Maroni, Johanna N. J. Weston, Hiroshi Kitazato, Alan J. Jamieson

**Affiliations:** ^1^ School of Biological Sciences and Oceans Institute The University of Western Australia Perth Western Australia Australia; ^2^ Biology Department Woods Hole Oceanographic Institution Woods Hole Massachusetts USA; ^3^ School of Marine Resources and Environment Tokyo University of Marine Science and Technology (TUMSAT) Tokyo Japan; ^4^ Danish Centre for Hadal Research Satellite Office at Tokyo University of Marine Science and Technology (TUMSAT) Tokyo Japan; ^5^ Minderoo‐UWA Deep‐Sea Research Centre, School of Biological Sciences and Oceans Institute The University of Western Australia Perth Western Australia Australia

**Keywords:** deep‐sea, DNA barcoding, mtDNA, *Notoliparis*, phylogeny, *Pseudoliapris*, species delimitation

## Abstract

The hadal zone, Earth's deepest oceanic region, is defined by distinct geological features and hosts a variety of endemic species, including the Liparidae Gill, 1861 (snailfishes). Ecological understanding of snailfishes dwelling at depths greater than 6000 m remains limited due to challenges in physical specimen collection and preservation. This study employs molecular tools to assess the phylogenetic relationships and distribution patterns of hadal snailfishes by analyzing three mitochondrial DNA markers (*16S, Cyt‐B, COI*) and incorporates 20 new specimens from the Japan and Tonga trenches (Pacific Ocean) and the Diamantina Fracture Zone (Indian Ocean). The phylogenetic hypotheses and species delimitation assessments were tested among a framework of six taxonomic units —*Pseudoliparis swirei* Gerringer and Linley, 2017, *Pseudoliparis belyaevi* Andriashev and Pitruk, 1993, *Notoliparis kermadecensis* Nielsen, 1964, *Notoliparis stewarti* Stein, 2016, and *Paraliparis selti* Linley, Gerringer, and Canto‐Hernández, 2022. The results revealed wider geographic distributions than previously thought, particularly for *Notoliparis* c.f. *stewarti*. Further, the molecular data support the hypothesis that *Notoliparis* Andriashev, 1975 should be treated as a subjective junior synonym of *Pseudoliparis* Andriashev, 1955. Our findings do emphasize the challenges and limitations of using DNA barcoding solely to distinguish closely related or recently diverged species. Together, this study advances the biogeographic understanding of hadal snailfishes and highlights the importance of expanding sampling efforts.

## Graphical abstract

Hadal snailfish are not endemic to single deep‐sea features, as evidenced by multiple documented range extensions of hadal snailfish species. Our data supports the recommendation that *Notoliparis* should be considered a subjective junior synonym of the genus *Pseudoliparis* and highlights the importance of expanding sampling efforts.

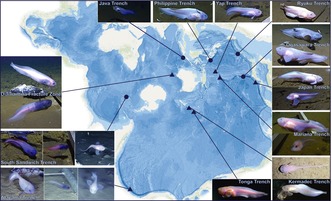

## 1 Introduction

The hadal zone (6000 m to ~11,000 m) represents one of the least explored regions, where subduction trenches, troughs, and fracture zones shape a unique and largely isolated ecosystem (Jamieson and Stewart 2021). These deep features host high levels of endemic species, facilitated through allopatric speciation and environmental selection (Jamieson et al. 2010). One such group that exhibits these endemic paradigms is the Liparidae Gill, 1861, or snailfishes. They are the most prominent fish family in this ecosystem—characterised by high hydrostatic pressure, near‐freezing temperatures, low food availability, and high seismic activity (Gerringer 2019; Jamieson, Linley, et al. 2021) – and are also among the most conspicuous megafaunal groups in the upper trenches (6000–8000 m; Gerringer 2019; Jamieson, Linley, et al. 2021). The evolutionary success of snailfishes has been attributed to several adaptations to high hydrostatic pressures, including high volumes of gelatinous tissues that enhance buoyancy and aid swimming efficiency (Gerringer, Popp, et al. 2017; Gerringer, Drazen, et al. 2017; Gerringer, Linley, et al. 2017; Gerringer et al. 2018; Priede 2018; Gerringer 2019; Jamieson, Linley, et al. 2021). Additionally, they exhibit elevated concentrations of the protein‐stabilising osmolyte trimethylamine n‐oxide (TMAO; Yancey 2020), but osmotic constraints associated with this adaptation are thought to physiologically limit them to upper hadal depths (Kelly and Yancey 1999; Linley et al. 2016; Yancey et al. 2014).

Most hadal depth subduction trenches surveyed host one or more snailfish species (Jamieson, Linley, et al. 2021), yet many of these records are based on baited camera observations rather than direct sampling. Assessing the full diversity of snailfish species within a trench can be challenging as some species cannot be distinguished by exterior morphology alone (Stein 2016). Additionally, determining species endemism between trenches partitioned by depths seemingly too shallow to cross remains unresolved due to the lack of physical samples relative to the number of known populations. Furthermore, several studies have reported the presence of eggs and larvae in snailfishes (Nielsen 1964; Able et al. 1984, 1986; Stein et al. 2001; Busby and Cartwright 2006; Takemura et al. 2010; Gardner et al. 2016; Stein 2016; Gerringer, Popp, et al. 2017; Gerringer, Drazen, et al. 2017; Gerringer, Linley, et al. 2017; Gerringer et al. 2018), yet details of their reproductive development and life history remain poorly understood. As a result, phylogenetic analyses of hadal snailfishes are limited, with existing studies (e.g., Orr et al. 2019; Linley et al. 2022) based on specimens from widely separated geographic regions and depths, making broader evolutionary patterns difficult to resolve.

Hadal snailfishes exhibit notable diversity, with species distributed across multiple genera and geographic regions, though many remain undescribed or have been identified only from imagery. Specifically, eight species of snailfish from approximately 13 hadal features worldwide are formally accepted as taxonomic units, yet up to 15 species have been estimated to exist (summarised in: Gerringer 2019; Jamieson, Linley, et al. 2021). Until the recent description of *Paraliparis selti* Linley, Gerringer, and Canto‐Hernández, 2022 from the Atacama Trench in the east Pacific Ocean (Linley et al. 2022), all described species belonged to the genera *Pseudoliparis* A. Andriashev 1955, *Notoliparis* Andriashev, 1975, and *Careproctus* Krøyer, 1862 (Linley et al. 2016; Stein 2016; Gerringer, Popp, et al. 2017; Gerringer, Drazen, et al. 2017; Gerringer, Linley, et al. 2017; Gerringer 2019; Jamieson, Linley, et al. 2021), with *Pseudoliparis* spp. restricted to the trenches in the North Pacific, *Notoliparis* spp. inhabiting trenches in the Southern Hemisphere, and *Careproctus sandwichensis* known from the South Sandwich Trench (Andriashev and Stein 1998). In addition to these two known genera, other morphotypes such as the ‘Ethereal Morph’ from the Mariana and Japan trenches in the north Pacific Ocean (Linley et al. 2016; Jamieson et al. 2023), the South Sandwich Trench in the Southern Ocean (Jamieson, Stewart, et al. 2021), and the Atacama Trench in the South Pacific (Jamieson, Linley, et al. 2021) have only been recorded via video and belong to uncertain and in some cases likely undescribed genera.

Collecting and preserving hadal snailfish specimens present significant challenges, often resulting in poor‐quality samples that limit taxonomic and ecological analyses. Hadal snailfishes are typically described from a single or few specimens, which frequently suffer from physical degradation due to prolonged or inadequate preservation (e.g., Stein et al. 1991; Stein 2005). When brought to the surface, a specimen undergoes rapid physiological changes as it experiences extreme differences in pressure (upwards of 600 bar) and temperature (upwards of 20°C), leading to melting‐like deterioration of its gelatinous tissues (Gerringer 2019). This degradation can obscure key anatomical features such as the cephalic sensory pores (e.g., Stein 2016), complicating species identification. Fixation and preservation processes also modify specimens, reducing their reliability for comparative studies. Combined with chronic under‐sampling of the hadal zone (Weston and Jamieson 2022), these factors contribute to the limited understanding of hadal snailfishes diversity, inter‐trench connectivity, population structure, and demography (Linley et al. 2016; Wang et al. 2019). Despite these preservation challenges, certain morphological characters remain crucial for identifying and classifying hadal snailfishes. Specifically, taxonomic analyses rely on counts of fin rays, vertebrae, and internal structures, such as the pectoral girdle and tooth morphology. The presence or absence of structures is also diagnostically important, such as the pelvic disk and pseudobranchia, which are less prone to degradation during collection (Stein 2012, 1975; Kido 1988; Knudsen et al. 2007; Duhamel et al. 2010; Orr et al. 2019; Kai et al. 2020). Despite the limitations of morphological data, these core traits are key for species identification.

Phylogenetic analyses have further refined liparid classifications, revealing polyphyletic groupings within *Careproctus* and *Paraliparis* and suggesting that *Notoliparis* and *Pseudoliparis*, despite their geographic separation, may be closely related both morphologically and genetically (Gerringer, Popp, et al. 2017; Gerringer, Drazen, et al. 2017; Gerringer, Linley, et al. 2017; Orr et al. 2019). Molecular data have also supplemented traditional morphological methods in species descriptions (Hebert et al. 2003; Pons et al. 2006; Thiele et al. 2022) and have been particularly powerful in cases where morphological variation is limited or difficult to discern (e.g., Payo et al. 2013; Wilson et al. 2013; Fassio et al. 2020; Maroni et al. 2022). Among shallower‐water liparid species, sequence data have been utilized alongside morphological data to unravel intra‐ and interspecific evolutionary patterns (e.g., Knudsen et al. 2007; Rock et al. 2008; Orr et al. 2019). In contrast, minimal molecular data are available for hadal liparids within the public domain due to the rarity of physical specimens. Specifically, for the standard metazoan mitochondrial barcoding region, cytochrome oxidase I (*COI*), only six *Pseudoliparis* and five *Notoliparis COI* sequences are available within NCBI GenBank (Benson et al. 2012). Despite such small datasets, this information has successfully complemented morphological information among a few hadal snailfish that were examined. For example, due to the uncertainty of pore counts, *Pseudoliparis swirei* Gerringer and Linley, 2017 was assigned to the genus *Pseudoliparis* rather than *Notoliparis* based solely on divergence data (Gerringer, Popp, et al. 2017; Gerringer, Drazen, et al. 2017; Gerringer, Linley, et al. 2017). In another example, genetic data facilitated the formal placement of *Paraliparis selti* Linley, Gerringer, and Canto‐Hernández, 2022 within the *Paraliparis* phylogeny, which provided strong support for the independent radiation of these species into the hadal zone (Linley et al. 2022). While these studies highlight the value of molecular data in liparid classification, the scarcity of available sequences—especially for hadal species—continues to limit our understanding of their evolutionary relationships and geographic distribution.

To address this gap, this study investigated the geographic distribution of hadal snailfishes with newly collected specimens from the Japan and Tonga trenches (Pacific Ocean) and the Diamantina Fracture Zone (Indian Ocean). We explored the diversity of these newly collected specimens' using three mitochondrial DNA markers in conjunction with species delimitation tools and haplotype network analyses. The objectives of this study were to (i) generate sequence data for all newly collected hadal snailfish specimens, (ii) compare these sequences with previously published molecular datasets, (iii) test for additional, unknown species, (iv) explore the distribution patterns among these species, and (v) reflect on the current state of systematics and biogeography for hadal snailfishes. This paper also evaluates the effectiveness of three standardized barcoding gene fragments in relation to resolving hadal snailfish species relationships and assesses whether they should be reconsidered for future studies. Overall, this study aims to test the degree to which hadal snailfishes are restricted to individual hadal features and explores the biogeographical ranges of these snailfishes in a global context.

## 2 Materials and Methods

### 2.1 Specimen Collection and Preservation

**FIGURE 1:**
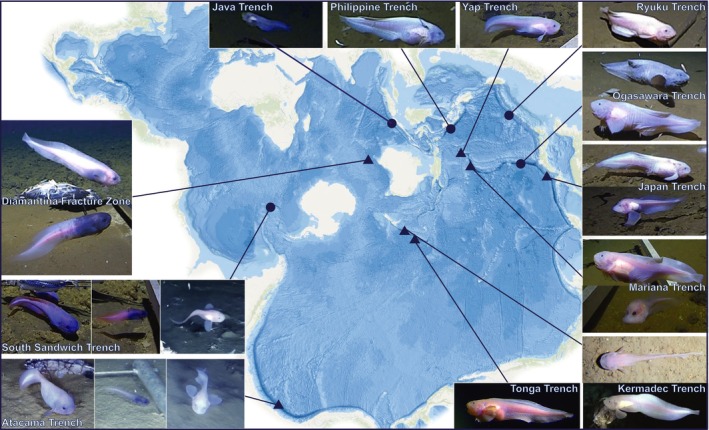
Spilhaus world projection showing most known hadal snailfish populations, with samples denoted with a triangle if they were collected and sequenced within this study. From the Diamantina Fracture Zone, both images show the two specimens that were caught and sequenced as a part of this study. All other sample images are derived from: Jamieson, Linley, et al. (2021); Jamieson, Stewart, et al. (2021) and the DSSV Pressure Drop ‘Five Deeps’ and ‘Ring of Fire’ expeditions. Java: Liparid sp. *indent*. 1‐JAV, 6439–7178 m, Philippines: Liparid sp. *indent*. 1‐PHT, 6227–7691 m, Yap: This study, 6903 m, Ryuku: ROF_JAPAN1, 5110–7339 m, Ogasawara, ROF_JAPAN1, 4534 m, Japan: ROF_JAPAN1&2, 4913–8022 m, Mariana: Top *P. swirei* , 6731‐7888 m, bottom Liparid sp. *indent*. 3‐MAR (‘the ethereal snailfish’), 7542 m, Kermadec: Top *N. kermadecensis* , 7199–7561 m, bottom *N. kermadecensis* , 6456–7554 m, Tonga: Liparid sp. *indent*. 1‐TT, 6848–7928 m, Atacama: Left Liparid sp. *indent*. 2‐PCT, 5329–6974 m, middle *P. selti*, 5920–7608 m, right Liparid sp. *indent*. 3‐PCT, 6520–7493 m, South Sandwich: Left Liparid sp. *indent*. 2‐SAND, 6044 m, middle *Paraliparis* sp. *indent*. 1‐SAND, 6044–6640 m, right Liparid sp. *indent*. 3‐SAND, 6640 m, Diamantina: this study, 6177 m.

Hadal snailfishes were recovered from two hadal trenches: Tonga Trench (SW Pacific; 6848–7272 m) in June 2019, and Japan Trench (NW Pacific Ocean; 7000–8022 m) from August and September 2022 on the DSSV *Pressure Drop* and one fracture zone: Diamantina Fracture Zone (SE Indian Ocean; 6177 m) in March 2022 on the *Pangea Ocean Explorer* (Figures 1, S1 and Table S1) using autonomous lander systems equipped with vertebrate traps (50 by 21 cm), baited with whole mackerel (Scombridae). Lander systems were also equipped with continuously recording high‐definition (HD) video cameras (IP Multi SeaCam 3105; Deep Sea Power and Light, San Diego, CA) and pressure and temperature sensors (SBE 49 FastCAT, SeaBird Electronics, Bellevue, WA) recording at 10 s intervals. Muscle tissue sub‐samples and fin clips were taken from fresh caught specimens and stored in 96%–100% ethanol. All deployment details and collection information are outlined in Table S1.

### 2.2 DNA Extraction, PCR Amplification, and DNA Sequencing

Total genomic DNA was extracted from 20 ethanol‐fixed sub‐samples using either a DNeasy Blood and Tissue Kit (Qiagen) according to the manufacturer's instructions or a magnetic‐bead‐based protocol (Oberacker et al. 2019). Three mitochondrial barcoding regions, 16S rRNA (*16S*), cytochrome b (*Cyt‐b*), and *COI* were amplified using published primer sets (Ward et al. 2005; Gerringer, Popp, et al. 2017; Gerringer, Drazen, et al. 2017; Gerringer, Linley, et al. 2017). Each PCR reaction contained 0.5 μM of each primer (forward and reverse primer), 1× AmpliTaqGold 360 Master Mix (Thermo Fisher Scientific), ~10 ng DNA template, and molecular grade (deionised) water to a final reaction volume of 25 μL. The following PCR conditions, adapted from Gerringer, Popp, et al. (2017); Gerringer, Drazen, et al. (2017); Gerringer, Linley, et al. (2017), were used for amplification of the *16S* fragment and the *Cyt‐b* fragment: 2 min at 95°C, 35 cycles of 30 s at 95°C, 30 s at 52°C, 1 min at 72°C, with a final extension time of 2 min at 72°C on a Veriti thermal cycler (Thermo Fisher Scientific). The primers used to amplify the *16S* gene fragment were 16S_liparids_F (5′‐CTA TTA ATA CCC CCA AAT ACC CC‐3′) and 16S_liparids_R (5′‐CGA TGT TTT TGG TAA ACA GGC G‐3′) as seen in Gerringer, Popp, et al. (2017); Gerringer, Drazen, et al. (2017); Gerringer, Linley, et al. (2017) and for *Cyt‐b*, the primers were Cytb_liparids_F (5′‐ATG GCA AGC CTA CGA AAA ACC CAC C‐3′) and Cytb_liparids_R2 (5′‐GGG TTA GTT GAG CCT GTT TCG TG‐3′; Gerringer, Popp, et al. 2017; Gerringer, Drazen, et al. 2017; Gerringer, Linley, et al. 2017). The PCR conditions for amplification of *COI* adapted from Ward et al. (2005) were: 4 min at 95°C, 35 cycles of 30 s at 94°C, 30 s at 50°C, 30 s at 72°C, with a final extension time of 10 min at 72°C. Primers for *COI* were Liparid_WardsF1 (5′‐TCG ACT AAT CAC AAA GAC ATT GGC AC‐3′) and Liparid_WardsR1 (5′‐TAA ACT TCG GGA TGG CCA AAG AAT CA‐3′) as seen in Ward et al. (2005). Single amplicons were confirmed using 96‐well E‐gels (Invitrogen) before being purified using AMPure XP paramagnetic beads using a 1.8:1 bead‐to‐sample ratio (Beckman and Coulter).

PCR products were prepared for sequencing reactions using a Thermo Fisher Scientific Applied Biosystems BigDye Cycle Sequencing Kit, cleaned using the CleanSEQ Dye‐Terminator Removal Protocol (Beckman Coulter), and sequenced on a 3730*xl* capillary sequencer (Thermo Fisher Scientific Applied Biosciences), either at the Australian Genome Research Facility (AGRF), Western Australia, or at Eurofins Genomics, Germany. All sequences were assembled and edited in Geneious Prime 2023.0.1 (Kearse et al. 2012). The sequence identity was confirmed using NCBI BLASTn (Altschul et al. 1990). Nucleotide sequences for *Cyt‐b* and *COI* were translated into amino acid sequences to check for the presence of stop codons.

Samples UWADSC004018 and UWADSC004019 were re‐sequenced using the same protocols as above to confirm their phylogenetic placement. This was required as the initial phylogenetic results deviated from the current literature regarding these species known distributions.

### 2.3 Additional Datasets

**FIGURE 2:**
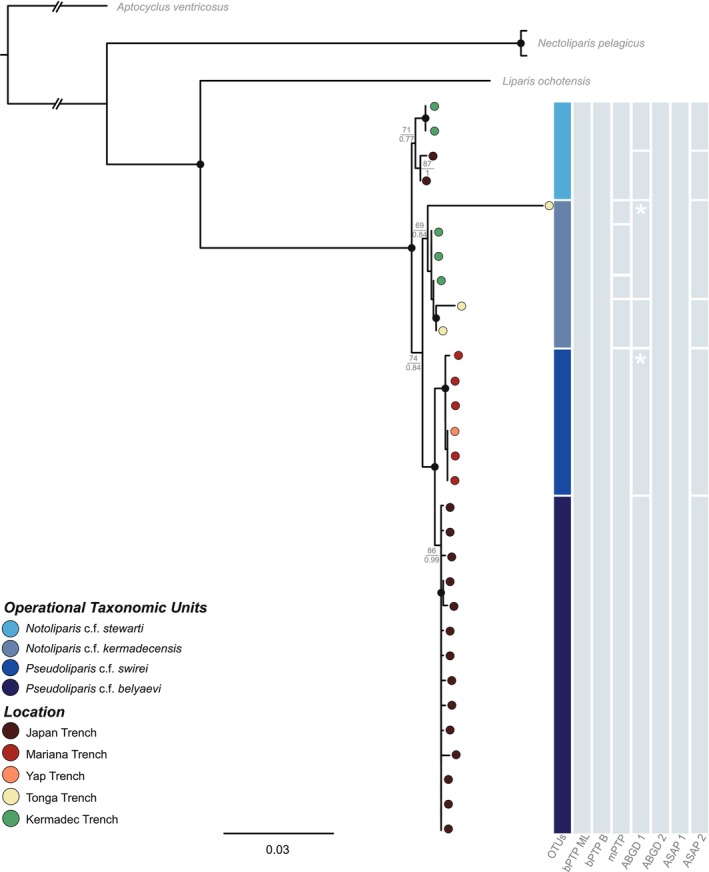
A maximum likelihood and Bayesian phylogeny of *Pseudoliparis* and *Notoliparis* using the concatenated genetic dataset. Nodes with ultrafast bootstrap (UF) support ≥ 90 and Bayesian posterior probability (PP) support ≥ 0.90 are denoted by a black circular node. All other support values are retained with the ML UF support values presented above and the Bayesian PP support values below the fraction midline. Boxes (right) represent the operational taxonomic units (OTUs) being tested, followed by the species delimitation results (bPTP, mPTP, ABGD, and ASAP). The asterix (*) represents results with clustered samples, but these samples are not directly next to each other within the tree. The circles right of the tree tips are colour‐coded to represent where that sample was collected from.

**FIGURE 3:**
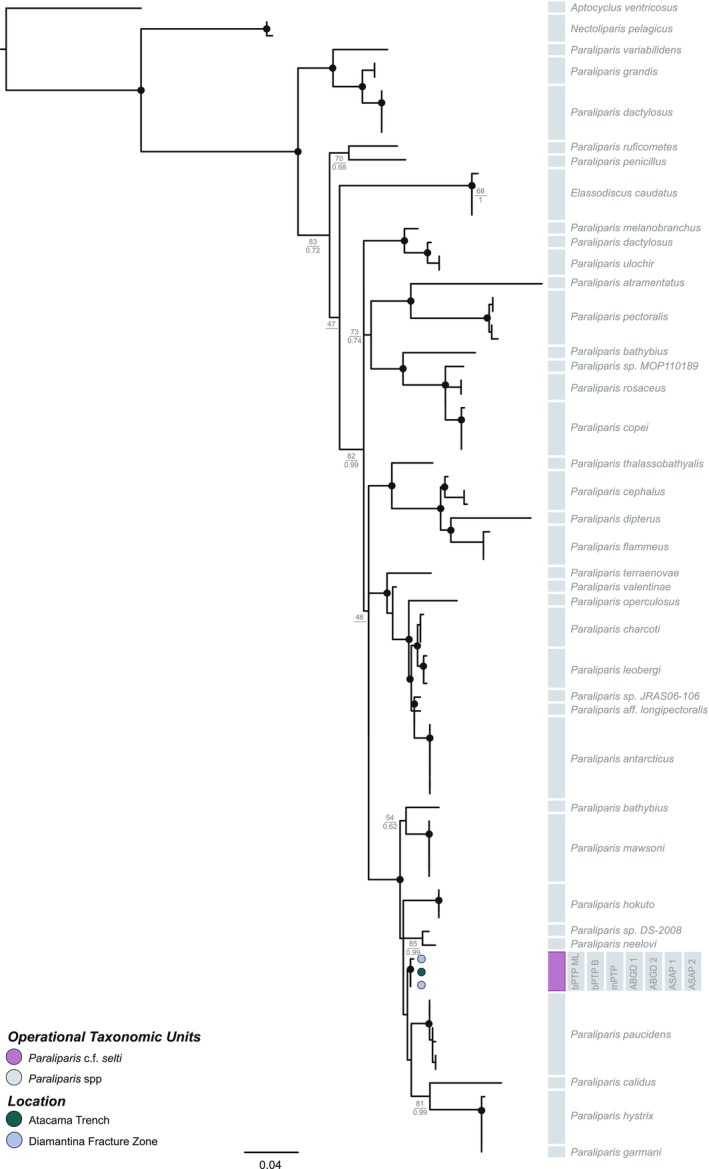
A *COI* (*n* = 84) maximum likelihood and Bayesian phylogeny of all *Paraliparis* species with *Paraliparis* c.f. *selti* indicated in purple. The tree is rooted with *Aptocyclus ventricosus* . Nodes with ultrafast bootstrap (UF) support ≥ 90 and Bayesian posterior probability (PP) support ≥ 0.90 are denoted by a black circular node.

One additional unpublished snailfish sequence from the Japan Trench from 6587 m (T.P. Satoh, NSMT Japan: 92445‐92447) was included, as well as all published sequences of *Pseudoliparis* (Gerringer, Popp, et al. 2017; Gerringer, Drazen, et al. 2017; Gerringer, Linley, et al. 2017; Mu et al. 2021) from the Mariana and Yap Trenches, *Notoliparis* (Gerringer, Popp, et al. 2017; Gerringer, Drazen, et al. 2017; Gerringer, Linley, et al. 2017) from Kermadec Trench, and *Paraliparis selti* (Linley et al. 2022) Atacama Trench, Pacific Ocean (Figures 2 and 3). Sequences of *Aptocyclus ventricosus* (smooth lumpfish), *Elassodiscus caudatus* (formally regarded to be *Paraliparis caudatus* ), *Nectoliparis pelagicus*, and *Liparis ochotensis* (Stein 1975; Miya et al. 2003; Knudsen et al. 2007; Steinke et al. 2009; Sim et al. 2020; Kai 2022) were incorporated as outgroup specimens (see Table S1 for all accession, sequence, and collection information).

### 2.4 Phylogenetic Analyses

Two main phylogenies were constructed to infer species boundaries among the study's specimens and all other relevant snailfish sequences. The first was a concatenated phylogeny including all sequenced specimens of *Pseudoliparis* and *Notoliparis* (*n* = 30), rooted with *Aptocyclus ventricosus* , a member of the Cyclopteridae family, which is considered the sister family to the Liparidae (Orr et al. 2019). The second was a *COI* phylogeny comprised of 34 *Paraliparis* species (*n* = 77 *COI* sequences). Additionally, single‐locus phylogenies were generated for all *Pseudoliparis* and *Notoliparis* sequences (herein referred to as the hadal snailfishes phylogenies) and all *Paraliparis* sequences (herein referred to as the *Paraliparis* phylogenies). Each dataset was aligned with the MAFFT plugin for Geneious (ver. 1.5.0, Katoh and Standley 2013) using default settings. Phylogenies were inferred for all datasets using both maximum likelihood (ML) and Bayesian approaches. Maximum likelihood phylogenies were reconstructed using IQ‐TREE2 (ver. 2.2.0, Trifinopoulos et al. 2016), and nodal support was assessed with 10,000 ultrafast bootstrap replicates (Hoang et al. 2018). For the ML analyses, the ‐m TEST (Kalyaanamoorthy et al. 2017) option in IQ‐TREE2 identified HKY + F for the *16S* dataset, TN + F + G4 for the *Cyt‐b* alignment, and HKY + F + I for the *COI* alignment as the best‐fit models chosen according to Bayesian Information Criterion (BIC). These best‐fit models were used as input to inform a partitioned complex maximum likelihood model for the concatenated alignment within IQ‐TREE2. Bayesian analyses were conducted using the Geneious Prime MrBayes plugin (ver. 3.2, Huelsenbeck and Ronquist 2001; Ronquist and Huelsenbeck 2003). Four independent Markov chain Monte Carlo (MCMC) runs were performed, and each run was composed of four heated chains (chain temperature = 0.2). Each analysis ran for 40,000,000 generations, with a burn‐in length of 4,000,000 and a sub‐sampling frequency of 10,000 under an HKY85 evolution model. Convergence of the runs was determined by examining the average standard deviation of split frequencies using Tracer (Rambaut et al. 2018) within the Geneious Prime MrBayes plugin.

Prior to species delimitation, all outgroup specimens were removed for model accuracy and pairwise distances (uncorrected p‐distances) or Tamura Nei (Tamura and Nei 1993; “TN93”) corrected genetic distances (most similar to the Hasegawa et al. 1985) were calculated using the *dist.dna* function from the R package Ape (ver. 5.6, Paradis and Schliep 2019) with either “raw” or “TN93” selected as the evolutionary model (Figure S2, Table 1). The base frequencies were specified for the TN93‐corrected genetic distance calculations (*16S*: *A* = 0.352, *C* = 0.224, *G* = 0.190, *T* = 0.234, *Cyt‐b*: *A* = 0.263, *C* = 0.239, *G* = 0.138, *T* = 0.359, *COI*: *A* = 0.233, *C* = 0.278, *G* = 0.176, *T* = 0.313).

**TABLE 1:** Corrected genetic distances for each genetic dataset (*16S, Cyt‐b* and *COI*).

	*N. c.f. kermadecensis*	*N. c.f. stewarti*	*P. c.f. swirei*	*P. c.f. belyaevi*	*P. c.f. selti*
*16S*					
*Notoliparis c.f. kermadecensis*	**0.000–0.507**				
*Notoliparis c.f. stewarti*	0.380–1.019	**0.000–0.508**			
*Pseudoliparis c.f. swirei*	0.764–1.408	0.891–1.019	**0.000–0.126**		
*Pseudoliparis c.f. belyaevi*	0.635–1.538	0.635–1.019	0.508–1.020	**0.000–0.508**	
*Paraliparis c.f. selti*	4.738–5.569	4.597–5.294	5.291–5.713	5.012–5.855	**0.000–0.253**
*Cyt‐b*					
*Notoliparis c.f. kermadecensis*	**0.000–1.683**				
*Notoliparis c.f. stewarti*	1.267–3.854	**0.000–0.261**			
*Pseudoliparis c.f. swirei*	1.707–3.878	2.131–3.013	**0.000–0.417**		
*Pseudoliparis c.f. belyaevi*	0.837–2.990	0.842–1.691	0.849–1.707	**0.000–0.849**	
*Paraliparis c.f. selti*	10.482–12.510	8.485–9.975	10.514–12.033	8.971–10.475	**0.000–0.842**
COI					
*Notoliparis c.f. kermadecensis*	**0.000–0.790**				
*Notoliparis c.f. stewarti*	0.782–1.757	**0.000–0.311**			
*Pseudoliparis c.f. swirei*	0.312–1.280	0.782–1.102	**0.000–0.155**		
*Pseudoliparis c.f. belyaevi*	0.470–1.613	0.782–1.416	0.155–1.102	**0.000–0.626**	
*Paraliparis c.f. selti*	6.423–7.285	6.423–7.306	6.761–7.113	6.928–7.294	**0.000–0.155**

*Note:* Intraspecific genetic distances are in bold, and interspecific values are below the diagonal.

### 2.5 Species Delimitation

The ML and Bayesian phylogenetic hypotheses were assessed in conjunction with one another to explore putative species boundaries, which were then further tested with delimitation methods. Rather than serving as absolute classifications, these boundaries were treated as working hypotheses. The taxonomic units relevant to this study*—Pseudoliparis swirei*, *Pseudoliparis belyaevi* Andriashev and Pitruk, 1993, *Notoliparis kermadecensis* Nielsen 1964, *Notoliparis stewarti* Stein 2016, and *Paraliparis selti—*were considered within this framework of phylogenetic hypotheses and species delimitation assessments.

Two tree‐based and two distance‐based species delimitation methods were applied to each dataset, and all analyses were run without outgroups. The first tree‐based approach was a Bayesian implementation of the Poisson Tree Processes model (bPTP; Zhang et al. 2013) using the default settings of 200,000 MCMC generations, thinning of 100, and a 0.1 burn‐in. The multi‐rate Poisson Tree processes analysis (mPTP; Kapli et al. 2017) was the second tree‐based method utilized. The two distance‐based methods were automatic barcode gap detection (ABGD; Puillandre et al. 2012) and assemble species by automatic partitioning (ASAP; Puillandre et al. 2021). For both ABGD and ASAP, the corrected genetic distances were imported for analysis, the default parameters were used, and the Kimura K80 (Kimura 1980) substitution model was selected with a transition/transversion rate of 2.0 (default). ABGD and ASAP outputs were investigated for a range of partitioning definitions to ensure all inter‐ and intraspecific diversity had been encapsulated within the species hypotheses. The primary partition within ABGD assumes that a single gap can be defined for an entire dataset. Yet among snailfishes, the barcoding gap between conspecific and congeneric values is poorly defined (Stein 2012; Orr et al. 2019); thus, both the ABGD initial partition 1 and partition 2 were investigated. Only the initial partition results were examined as these partitions generally represent species or operational taxonomic units (OTUs) rather than the population‐level diversity within the data (recursive partitions; Puillandre et al. 2012). When examining the results from ASAP for each dataset, the first and second ASAP delimitation hypotheses were considered as these partitions had low and thus better‐supported ASAP scores of between 1.00 and 3.00.

### 2.6 Haplotype Structure, Diversity, and Connectivity

To assess the geographic structure of haplotypes, single‐locus TCS (Clement et al. 2000) haplotype networks were constructed in PopART (ver. 1.7, Leigh and Bryant 2015) with a 95% probability threshold, and the levels of polymorphism were represented by segregating sites (*ss*) (Fu 1995). Locality data were overlaid, and seven hadal features were defined a priori (Figure 1). Haplotypic (*h*) and nucleotide diversity (*π*) (Nei 1987) indices were calculated using the R packages Pegas (ver. 1.2, Paradis 2010) and Ape (ver. 5.7‐1, Paradis and Schliep 2019).

## 3 Results

This study focused on newly acquired hadal snailfishes from Tonga Trench (SW Pacific; 6848–7273 m, *n* = 2), Japan Trench (NW Pacific Ocean; 6833–8022 m, *n* = 16), and the Diamantina Fracture Zone (SE Indian Ocean; 6177 m, *n* = 2) and placed them in the context of global hadal snailfish diversity. Comparative records included sequences from the Mariana Trench (NE Pacific Ocean; 6949–7652 m, *n* = 5), Yap Trench (NE Pacific Ocean; 6903 m, *n* = 1), Kermadec Trench (SW Pacific Ocean; 6456–7554 m, *n* = 5), and the Atacama Trench (East Pacific Ocean; 6714 m, *n* = 1) (Stein 2016; Gerringer, Popp, et al. 2017; Gerringer, Drazen, et al. 2017; Gerringer, Linley, et al. 2017; Mu et al. 2021; Linley et al. 2022). The final *16S* hadal snailfish alignment (1463 bp) consisted of 30 ingroup sequences, the *Cyt‐b* alignment (1007 bp) consisted of 29 ingroup sequences, and the *COI* (1399 bp) alignment consisted of 31 ingroup sequences. The *COI Paraliparis* phylogeny consisted of 34 *Paraliparis* species (*n* = 77, 487 bp), the *16S* phylogeny consisted of 8 *Paraliparis* species (*n* = 13, 1416 bp), and the *Cyt‐b* phylogeny consisted of 7 *Paraliparis* species (*n =* 10, 422 bp). All *Paraliparis* phylogenies also contained sequences of *Elassodiscus caudatus*. Overall, this study produced 20 new sequences, available on NCBI Genbank (*16S*: PP446523–PP446539, *Cyt‐b*: PP458984–PP459000, *COI*: PP446511–PP446522, PP447191–PP447195).

### 3.1 Species Delimitation

The newly collected specimens are most closely related to the described species *Pseudoliparis swirei, Pseudoliparis belyaevi, Notoliparis kermadecensis, Notoliparis stewarti*, and *Paraliparis selti*. Given the limited genetic resolution of this study, the species delimitation results (presented below) and the absence of additional diagnostic data, these phylogenetic placements should be interpreted with caution. Without broader genomic data or complementary morphological assessments, the genetic markers used here may not provide sufficient resolution to formally classify our specimens. Therefore, no formal taxonomic assignment is made for these newly collected specimens. Specifically, and for the purposes of this study, the specimens collected from Japan Trench that are genetically similar to *P. swirei* will be referred to as *Pseudoliparis* c.f. *swirei*, specimens collected from Japan Trench that are genetically similar to *P. belyaevi* will be referred to as *Pseudoliparis* c.f. *belyaevi*, samples similar to *N. kermadecensis*, collected from Tonga Trench will be referred to as *Notoliparis* c.f. *kermadecensis*, samples closely related to *N. stewarti* from Japan Trench will be referred to as *Notoliparis* c.f. *stewarti*, and the Diamantina Fracture Zone samples that are genetically similar to *P. selti* will be referred to as *Paraliparis* c.f. *selti* to acknowledge their affinity while recognizing the need for further morphological and genetic validation. Given this, all tree‐based methods suggested that the new samples from Japan Trench represent *Pseudoliparis* c.f. *belyaevi* (6833–8022 m) and *Notoliparis* c.f. *stewarti* (7211–7453 m), *Notoliparis* c.f. *kermadecensis* was collected from Tonga Trench (6848–7273 m; Figure 2) and that the two individuals from the Diamantina Fracture Zone most closely relate to *Paraliparis* c.f. *selti* (6177 m; Figure 3).

Across both datasets, the ML and Bayesian phylogenies had high nodal support (> 95/0.95) for terminal clusters and mostly high support across the interior nodes (> 80/0.8), apart from the *Pseudoliparis* c.f. *belyaevi* cluster where the internal support values were mostly low (< 60/0.6; Figure 2). Within the concatenated hadal snailfish alignment, the proportion of invariable sites was 0.627, the base frequencies were unequal, and the rates for the six substitution types were *AC* = 4.669, *AG* = 29.672, *AT* = 4.669, *CG* = 1.000, *CT* = 29.672, *GT* = 1.000 (unequal transition/transversion rates). Within the *Paraliapris COI* alignment, the proportion of invariable sites was 0.515, the base frequencies were unequal, and the rates for the six substitution types were *AC* = 1.000, *AG* = 17.952, *AT* = 0.409, *CG* = 0.409, *CT* = 5.947, and *GT* = 1.000 (unequal transition/transversion rates).

The “TN93” corrected intraspecific genetic distances from across all datasets ranged from 0.000%–0.417% for *Pseudoliparis* c.f. *swirei*, 0.000%–0.849% for *Pseudoliparis* c.f. *belyaevi*, 0.000%–1.683% for *Notoliparis* c.f. *kermadecensis*, 0.000%–0.508% for *Notoliparis* c.f. *stewarti*, and 0.000%–0.842% for *Paraliparis* c.f. *selti* (Table 1). Interspecific distances were also small across species of *Pseudoliparis* and *Notoliparis*, ranging from 0.155% to 1.757%, except for *P. c.f. swirei* and *N*. c.f. *stewarti* having an interspecific distance maximum of 3.013% within the *Cyt‐b* dataset. Larger interspecific distances were seen between species of *Pseudoliparis* and *Notoliparis* when compared to *Paraliparis* c.f. *selti*, ranging from 4.597% to 12.510%. This is also illustrated in Figures 2 and 3, where the 3% and 4% divergences indicate relatively small evolutionary distances between taxa.

None of the species delimitation methods utilized within this study rendered a well‐supported species hypothesis (Figures 2, 3 and [Link], [Link]). Overall, 46% of the delimitation results collapsed the representatives of *Pseudoliparis* and *Notoliparis* together and separated them from *Paraliparis* c.f. *selti* (Figure S6). The bPTP analysis showed the highest evidence for over‐splitting, delimiting between two and 22 species, whereas mPTP recovered the smallest range of species compared to all other delimitation methods (delimited between one and seven species). The ABGD initial partitions delimited between two and 14 species, ASAP's 1st partition converged on two species across all datasets, and ASAP's 2nd partition delimited between four and ten species.

### 3.2 Haplotype Structure and Diversity Estimates

**FIGURE 4:**
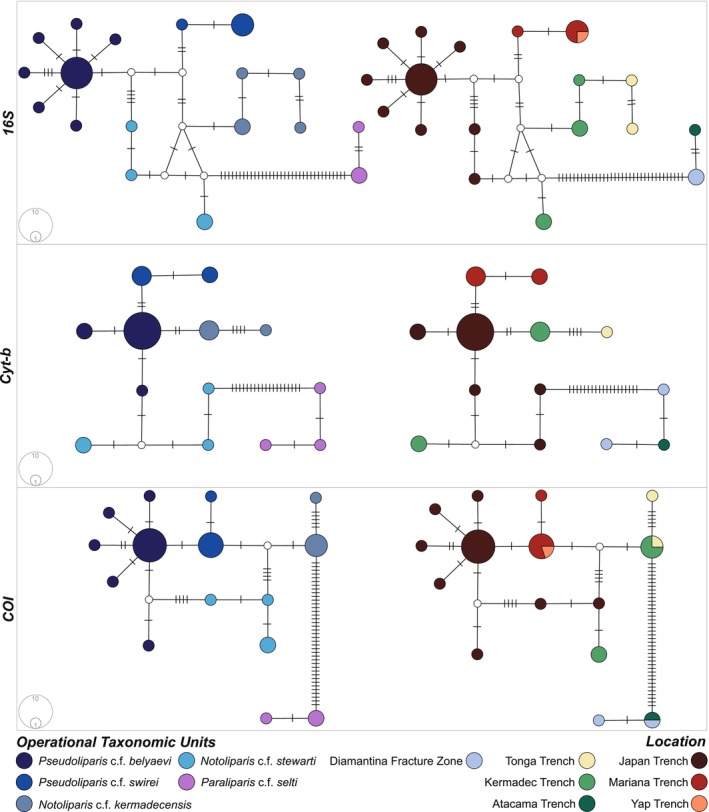
TCS haplotype networks for all *Paraliparis, Pseudoliparis*, and *Notoliparis* species examined within this study. Top: *16S*, middle: *Cyt‐b*, and bottom: *COI*. Left: The colors within the network represent the operational taxonomic units exemplified here; right: Colors represent the location from which corresponding samples were collected. The area of each circle is proportional to the frequency of the haplotype, and the nodes represent unsampled or extinct haplotypes.

The presence of a few dominant haplotypes and many low frequency closely related haplotypes was reflected with high haplotypic diversity (*16S*: *h* = 0.9204, *Cyt‐b*: *h* = 0.9149, *COI*: *h* = 0.8911), and low nucleotide diversity values (*16S*: *π* = 0.01384, *Cyt‐b*: *π* = 0.02617, *COI*: *π* = 0.01551; Table 2, Figure 4). The *16S* network consisted of 23 haplotypes, with one *Pseudoliparis* c.f. *swirei* shared haplotype determined between the Mariana and Yap trenches and five unknown nodes. The *Cyt‐b* network consisted of 14 haplotypes, all private with one unknown node, and the *COI* network contains 17 haplotypes, three of which were shared between *Pseudoliparis* c.f. *swirei* at the Mariana and Yap trenches, *Paraliparis* c.f. *selti* at the Diamantina Fracture Zone and Atacama Trench, and *Notoliparis* c.f. *kermadecensis* at the Kermadec and Tonga trenches.

**TABLE 2:** Haplotype and Nucleotide diversity indices and number of segregating sites for each species and genetic dataset.

Dataset	Taxonomic unit	*n*	Nucleotide diversity (*π*)	Haplotype diversity (*h*)	Segregating sites
*16S*	*N. c.f. kermadecensis*	5	0.0005	0.4	1
	*N. c.f. stewarti*	4	0.00315	0.83333	4
	*P. c.f. belyaevi*	14	0.0014	0.6923	8
	*P. c.f. swirei*	5	0.0005	0.4	1
	*P. c.f. selti*	3	0.00168	0.66666	2
*COI*	*N. c.f. kermadecensis*	5	0.0031	0.4	5
	*N. c.f. stewarti*	4	0.0018	0.83333	2
	*P. c.f. belyaevi*	14	0.00155	0.60439	7
	*P. c.f. swirei*	6	0.00051	0.33333	1
	*P. c.f. selti*	3	0.00103	0.66666	1
*Cytb*	*N. c.f. kermadecensis*	4	0.00829	0.5	4
	*N. c.f. stewarti*	4	0.00793	0.83333	4
	*P. c.f. belyaevi*	14	0.00174	0.68131	4
	*P. c.f. swirei*	5	0.0019	0.6	1
	*P. c.f. selti*	3	0.00423	1	2

## 4 Discussion

Hadal snailfishes have long been presumed to be highly endemic to a single hadal feature (Jamieson, Linley, et al. 2021); however, the multi‐locus sequence data for 30 specimens from five hadal trenches and the *COI Paraliparis* analysis containing specimens from one hadal feature and one fracture zone both suggest that their distributions could be more expansive. In compiling a census of all fish from depths exceeding 5000 m derived from 184 lander deployments, Jamieson, Linley, et al. (2021) used taxonomic nomenclature consistent with Sigovini et al. (2016) and identified 15 geographically tagged populations from video footage, with just three representing known species based on physical specimens. The results from this study allow us to refine this census. For example, the dominant species in the Tonga Trench (Liparid sp. *indet*. 1‐TT) is here documented as *Notoliparis* c.f. *kermadecensis*, which is the same species as that in the neighbouring Kermadec Trench. This is plausible given these trenches are only separated by a single seamount, the Osbourn Seamount (Gnibidenko et al. 1985). A similar scenario is *Pseudoliparis* sp. from the Yap Trench (Mu et al. 2021), reported here as *Pseudoliparis* c.f. *swirei*. The distance between the two trenches is only 95 km and shallows to just over 5000 m, a boundary that should be crossable given the known depth ranges of these species (Jamieson et al. 2020; Jamieson and Stewart 2021). This is also similar to *Pseudoliparis* c.f. *belyaevi* being found in both the Japan and Kurile‐Kamchatka trenches (A. Andriashev 1955). Combined, these findings show that species of snailfish that appear to be endemic to a single trench inhabit neighbouring trenches.

Another range extension that questions species‐ and genus‐level taxonomic classifications is the documentation of *Notoliparis* c.f. *stewarti* in Japan Trench. That is a geographic range extension from the Kermadec Trench (Stein 2016), some 8600 km, and the first record of a *Notoliparis* in the Northern Hemisphere. This challenges the idea that *Notoliparis* are restricted to the Southern Hemisphere and *Pseudoliparis* occur only in the Northern Hemisphere.

This study also found that both snailfish morphotypes from 6177 m in the Diamantina fracture zone are most closely related to *Paraliparis selti*, originally described from the Atacama Trench. Orr et al. (2019) suggested that the *COI* gene may not be effective for distinguishing among *Paraliparis* species, as they observed a lack of sequence divergence despite described morphological differences across the genera. As our study lacks additional molecular markers, our findings are limited by the available data. However, they confirm the presence of a *Paraliparis* species in this deep‐sea feature of the East Indian Ocean, highlighting the need for further research to verify its morphological identity or formally describe it as a new species.

### 4.1 No Defined Barcoding Gap

The lack of resolution among species delimitation analyses may be attributed to the recent radiation of these species (Rabosky et al. 2013), a hypothesis corroborated by both the low genetic divergence and the small number of mutations separating observed haplotypes. Snailfishes are one of the fastest‐evolving marine fish clades (Rabosky et al. 2018) having diverged from lumpfishes (Cyclopteridae Bonaparte, 1831) approximately 19.05–39.6 Mya during the Ogliocene–Miocene period (Dettai and Lecointre 2005; Smith and Wheeler 2004). Similar difficulties in species delimitation have been documented across other teleosts (bony fishes), such as *Aphanius* (Esmaeili et al. 2020), where 52.5%–65% of nominal species could not be clearly delimited, and *Elops* (de Sousa et al. 2022) where seven accepted species were grouped into a single cluster.

Snailfishes also exhibit a noticeable lack of a barcoding gap between congeneric and conspecific values (e.g., Meyer and Paulay 2005; Stein 2012; Orr et al. 2019). In this study, intraspecific distances were particularly high for *Paraliparis* c.f. *selti* but marginal between species of *Pseudoliparis* and *Notoliparis*. These findings align with Ward (2009), who analyzed 4636 *COI* genetic distances from 1088 freshwater and marine fish species and found that even at 0.5%–1% divergence, there was a 30% chance of species identification, increasing to 90% at 2% divergence. Similarly, Smith et al. (2012) reported zero to shallow genetic divergence among Ross Sea liparids, and Gerringer, Popp, et al. (2017); Gerringer, Drazen, et al. (2017); Gerringer, Linley, et al. (2017) found congeneric distances of 0.6%–1.4% at *Cyt‐b* and 0.7%–1% at *16S* among *Pseudoliparis* and *Notoliparis*. Orr et al. (2019) further analyzed phylogenetic relationships among 18 liparid genera, reporting overall divergences ranging from 0% to 21%, with a mean of 11.5%. However, within *Pseudoliparis* and *Notoliparis*, divergences were only 0.2%–0.8%, and the distance between these two deep‐sea genera was the smallest reported (0.5%), suggesting close evolutionary relationships (Orr et al. 2019).

### 4.2 Taxonomic Recommendations

Previous work by Gerringer, Popp, et al. (2017); Gerringer, Drazen, et al. (2017); Gerringer, Linley, et al. (2017) and Orr et al. (2019) has suggested that *Notoliparis* may be derived members from *Pseudoliparis* with Orr et al. (2019) suggesting that their distinction at the genus level may not reflect true evolutionary relationships, classifying the two genera within a clade along with *Careproctus crozetensis* (“Clade B: Pseudoliparia”). Morphologically, *Notoliparis* has been distinguished from *Pseudoliparis* based on the presence of additional postcoronal and temporal cephalic pores (Andriyashev and Pitruk 1993). However, given the fragility of snailfishes skin and the tendency of these pores to close or become damaged during sampling, their reliability as a diagnostic trait is questionable. This is further highlighted by the description of *Pseudoliparis swirei* from the Mariana Trench (Gerringer, Popp, et al. 2017; Gerringer, Drazen, et al. 2017; Gerringer, Linley, et al. 2017), where cephalic pore counts were found to be an inconsistent distinguishing feature. Overall, *Pseudoliparis* and *Notoliparis* overlap in many meristic characters, making their separation based solely on morphological traits difficult. As a result, some researchers have proposed synonymising *Notoliparis* and *Pseudoliparis*.

We echo this recommendation that *Notoliparis* Andriashev, 1975 should be considered as a junior synonym of *Pseudoliparis* A. Andriashev 1955, as initially suggested by Gerringer, Popp, et al. (2017); Gerringer, Drazen, et al. (2017); Gerringer, Linley, et al. (2017), and subsequently by Orr et al. (2019), Blanton et al. (2022), and Linley et al. (2022). Our results depict a non‐reciprocal monophyly within the two currently hypothesized genera. While *Notoliparis* c.f. *stewarti* serves as the basal lineage, the internal clade contains *Notoliparis* c.f. *kermadecensis, Pseudoliparis* c.f. *swirei*, and *Pseudoliparis* c.f. *belyaevi* with both *Pseudoliparis* species branching from *Notoliparis* c.f. *kermadecensis*. This recommendation would lead to multiple nomenclatorial changes.

To reassess the genus‐level placement of these snailfishes and resolve their taxonomic uncertainty, an integrative approach combining molecular, morphological, and ecological data is necessary. High‐throughput sequencing techniques, such as ultra conserved elements (UCEs) or restriction‐site associated DNA sequencing (RADseq), should be employed to generate robust phylogenies that clarify evolutionary relationships and test the monophyly of *Notoliparis* and *Pseudoliparis*. Morphological re‐evaluations should focus on a comprehensive set of characters beyond cephalic pore patterns, incorporating skeletal and myological traits that may provide more stable diagnostic features. Standardised imaging techniques, such as micro‐computed tomography (micro‐CT), can facilitate detailed anatomical comparisons while minimising the risk of specimen degradation. Additionally, the inclusion of ecological and depth‐related data may offer insights into potential adaptive divergence between these genera. Overall, a multidisciplinary framework integrating these methodologies will provide a more rigorous and holistic reassessment of the genus‐level classification within hadal snailfishes.

### 4.3 Geological Constraints for Species Distributions

In the Pacific Ocean, two‐thirds of all hadal trenches are situated in the western region and can be broadly categorized into two groups: those located in the northwest and those found in the southwest. Recent tectonic research indicates that around 52 Mya, subduction initiation commenced across the western Pacific plate margin in both the northern hemisphere and through the southern hemisphere (Reagan et al. 2023). Models also suggest that within the first 2 Mya after subduction initiation, the leading edge of the Pacific plate rapidly sunk to present‐day depths (Maunder et al. 2020; Reagan et al. 2023). This relatively rapid formation, followed by prolonged geological stability, may have provided a long‐term refuge for deep‐sea taxa, allowing for the continuous occupation of these extreme environments.

The relative stability of these trenches over geological time may explain the lack of speciation at the trench level. While early environmental shifts likely facilitated colonization and the establishment of trench‐specific populations, the absence of significant recent topographical or oceanographic changes may have limited opportunities for isolation‐driven divergence. Additionally, more recent tectonic interactions, including trench‐trench connectivity and potential tectonic collisions, may have facilitated gene flow between populations, further reducing the likelihood of speciation.

In the Yap and Mariana trenches (western Pacific), the lack of speciation between the two populations may be explained by the relatively shallow partition between the two trenches. The southernmost tip of the Mariana Trench, located near its intersection with the Caroline Ridge, lies approximately 95 km east of the easternmost tip of the Yap Trench, with the two trenches separated by a minimum depth of around 5330 m (Jamieson and Stewart 2021). Given this relatively shallow divide and the proximity of these deep‐sea environments, it is possible that connectivity between snailfish populations has been maintained, facilitating gene flow and limiting divergence.

Similarly, in the Kermadec and Tonga trenches (south Pacific), the lack of speciation between the two populations may be explained as the physical partition between the two trenches is the Osborn Seamount, the most westerly seamount in the Louisville Seamount Chain (LSM; Lonsdale 1988). The LSM is believed to have subducted beneath the Indo‐Australian plate in the last ~4 Mya (Timm et al. 2013). As the chain subducts, the hadal portion of these trenches will go through cycles of partitioning and re‐opening, which may provide sufficient opportunity for both populations to remain genetically similar.

## 5 Conclusions

This study revealed that multiple snailfish species have broader geographic distributions than previously anticipated and represents a substantial step forward in the field regarding the number of hadal snailfishes collected to date, demonstrating the value of increasing geographic scope in sampling. For the first time, *Pseudoliparis* and *Notoliparis* species have been putatively reported as sympatric within the Japan Trench. Also, *Notoliparis* c.f. *kermadecensis* has been reported from Tonga Trench and *Pseudoliparis* c.f. *swirei* has been documented from Yap Trench. These range extensions allow us to discuss the origin of deep‐sea snailfishes which, in turn, allows us to evaluate evolution across geological timescales. As the generation of robust phylogenies is crucial in our race against the current biodiversity crisis, future work could next incorporate nuclear markers or capture methods to gain the phylogenomic resolution necessary to unravel these complex taxonomic questions.

## Author Contributions

**Paige J. Maroni:** conceptualization (lead), data curation (lead), formal analysis (lead), investigation (lead), methodology (lead), project administration (lead), resources (equal), visualization (lead), writing – original draft (lead), writing – review and editing (equal). **Johanna N. J. Weston:** conceptualization (supporting), investigation (supporting), methodology (supporting), resources (equal), writing – review and editing (equal). **Hiroshi Kitazato:** funding acquisition (equal), project administration (equal), resources (equal), writing – review and editing (equal). **Alan J. Jamieson:** funding acquisition (equal), project administration (equal), resources (equal), supervision (equal), writing – review and editing (equal).

## Conflicts of Interest

The authors declare no conflicts of interest.

## Supporting information


**Figure S1.** Field images showing the recent samples collected from the Japan Trench (outlined in red) and the Diamantina fracture zone (outlined in pink). Scale bar: 2 cm.

**Figure S2.** Pairwise distances (uncorrected p‐distances) or Tamura Nei (1993) (TN93) corrected genetic distances were calculated from the hadal liparid single loci datasets using the *dna.dist* function in the R.cran (ver. 4.2.1, cite) package Ape (ver.5.6‐2, cite) with either “raw” or “TN93” selected as the evolutionary model.

**Figure S3.** A maximum likelihood and Bayesian phylogeny of *Paraliparis* c.f. *selti, Pseudoliparis* and *Notoliparis* using the mtDNA gene region, *16S*. The tree is rooted with *Aptocyclus ventricosus* and an additional outgroup, *Liparis ochotensis* is shown. Nodes with ultrafast bootstrap (UF) support values of 95 or higher and Bayesian posterior probability (PP) support values of 0.95 or higher are denoted by a black circular node shape. All other support values are retained with the ML UF support values presented above and the Bayesian PP support values below the fraction midline. Boxes represent the operational taxonomic units (OTUs) followed by the partition results for all species delimitation analyses (bPTP, mPTP, ABGD and ASAP). Shapes presented within the boxes under species delimitation results represent where a delimitation result has clustered samples, but these samples are not directly next to each other within the phylogenetic tree.

**Figure S4.** A maximum likelihood and Bayesian phylogeny of *Paraliparis* c.f. *selti, Pseudoliparis* and *Notoliparis* using the mtDNA gene region, *Cyt‐b*. The tree is rooted with *Aptocyclus ventricosus* and an additional outgroup, *Liparis ochotensis* is shown. Nodes with ultrafast bootstrap (UF) support values of 95 or higher and Bayesian posterior probability (PP) support values of 0.95 or higher are denoted by a black circular node shape. All other support values are retained with the ML UF support values presented above and the Bayesian PP support values below the fraction midline. Boxes represent the operational taxonomic units (OTUs) followed by the partition results for all species delimitation analyses (bPTP, mPTP, ABGD and ASAP).

**Figure S5.** A maximum likelihood and Bayesian phylogeny of *Paraliparis* c.f. *selti, Pseudoliparis* and *Notoliparis* using the mtDNA gene region, *COI*. The tree is rooted with *Aptocyclus ventricosus* and an additional outgroup, *Liparis ochotensis* is shown. Nodes with ultrafast bootstrap (UF) support values of 95 or higher and Bayesian posterior probability (PP) support values of 0.95 or higher are denoted by a black circular node shape. All other support values are retained with the ML UF support values presented above and the Bayesian PP support values below the fraction midline. Boxes represent the operational taxonomic units (OTUs) followed by the partition results for all species delimitation analyses (bPTP, mPTP, ABGD and ASAP). Shapes presented within the boxes under species delimitation results represent where a delimitation result has clustered samples, but these samples are not directly next to each other within the phylogenetic tree.

**Figure S6.** A comparison of the species delimitation results determined per genetic dataset. For the concatenated dataset, bPTP recovered two taxonomic units, mPTP yielded 7 species‐level groups, species delimitation based on the “TN93” corrected‐genetic distances using ABGD detected between six (initial partition 1, *p* = 0.001) and two (initial partition 2, *p* = 0.00167) units using the K80, TS/TV = 2.0 model and ASAP delimited between two (ASAP‐score = 1.00) and seven (ASAP‐score = 2.00) species, also using the K80, TS/TV = 2.0 model. For 16S, bPTP recovered between two (maximum likelihood solution) and 21 (highest Bayesian support solution) taxonomic units, mPTP yielded one species‐level group, species delimitation based on the “TN93” corrected‐genetic distances using ABGD detected between nine (initial partition 1, *p* = 0.001) and two (initial partition 2, *p* = 0.00167) units using the K80, TS/TV = 2.0 model and ASAP delimited between two (ASAP‐score = 1.00) and eight (ASAP‐score = 3.00) species, also using the K80, TS/TV = 2.0 model. For Cyt‐b, bPTP yielded two taxonomic units, mPTP delimited four species overall, ABGD detected between 14 (initial partition 1, *p* = 0.001, initial partition 2, *p* = 0.00167) species units using the K80, TS/TV = 2.0 and ASAP recovered between 2 (ASAP‐score = 1.00) and 10 (ASAP‐score = 2.00) species, also using the K80, TS/TV = 2.0 model and based on the “TN93” corrected‐genetic distances. For COI, bPTP recovered between three (maximum likelihood solution) and 21 taxonomic units (highest Bayesian support solution), mPTP delimited 3 species overall, for ABGDs initial partition 1 and 2, the program detected 2 species units (initial partition 1, *p* = 0.001, initial partition 2, *p* = 0.00167) using the K80, TS/TV = 2.0 and ASAP recovered between 2 (ASAP‐score = 1.00) and 4 (ASAP‐score = 2.50) species, also using the “TN93” corrected‐genetic distances and the K80, TS/TV = 2.0 model.

**Table S1.** Specimen metadata with the collection location, depth, year collected, method of collection, and the associated Genbank numbers per loci.

## Data Availability

All sequence data can be accessed via Genbak: *16S*: PP446523–PP446539, *Cyt‐b*: PP458984–PP459000, *COI*: PP446511–PP446522, and PP447191–PP447195. All supporting information and all figures are included in the publication or can be accessed via Dryad https://doi.org/10.5061/dryad.tmpg4f55z.
