# Supplementary material for: Hadal Snailfishes (Teleostei: Liparidae) Extend Across Multiple Trenches: Molecular Insights and Implications for Taxonomic Nomenclature
Source: Ecol Evol. 2025 Sep 29;15(10):e71779. doi: 10.1002/ece3.71779 (PMC12479125; doi:10.1002/ece3.71779)
Supplement: Supplementary file 1 — Figure S1. Field images showing the recent samples collected from the Japan Trench (outlined in red) and the Diamantina fracture zone (outlined in pink). Scale bar: 2 cm. [file ECE3-15-e71779-s003.pdf]

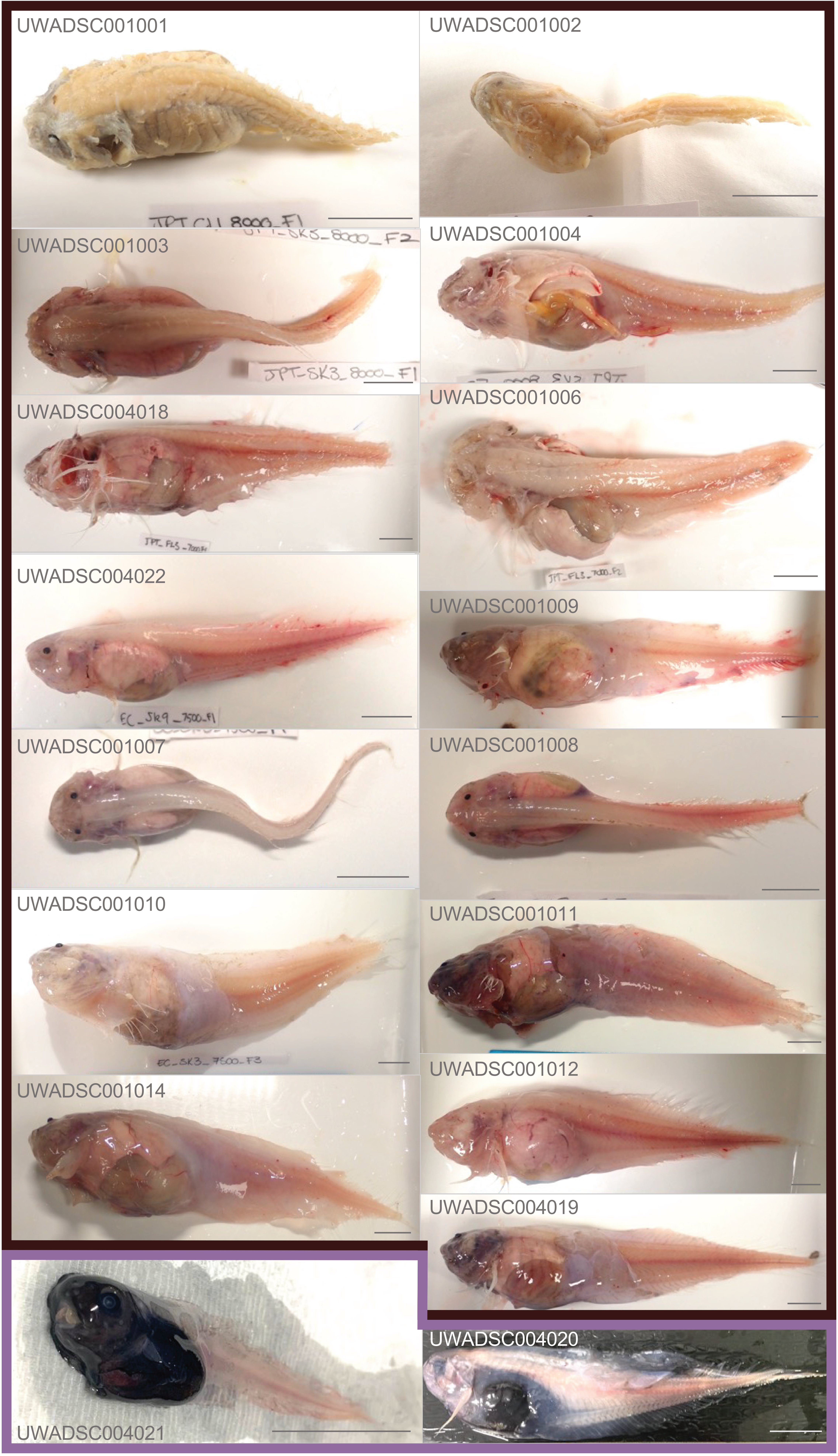
UWADSC001001
UWADSC001002
UWADSC001003
JPT-SK3_8000_F1
UWADSC001004
UWADSC004018
UWADSC001006
UWADSC004022
EC_SK9_7500_F1
UWADSC001009
UWADSC001007
UWADSC001008
UWADSC001010
EC_SK3_7500_F3
UWADSC001011
UWADSC001014
UWADSC001012
UWADSC004019
UWADSC004020
UWADSC004021
