# Supplementary figures and images for: Hadal Snailfishes (Teleostei: Liparidae) Extend Across Multiple Trenches: Molecular Insights and Implications for Taxonomic Nomenclature
Source: Ecol Evol. 2025 Sep 29;15(10):e71779. doi: 10.1002/ece3.71779 (PMC12479125; doi:10.1002/ece3.71779)

**“raw”**

**“TN93”**

**16S**

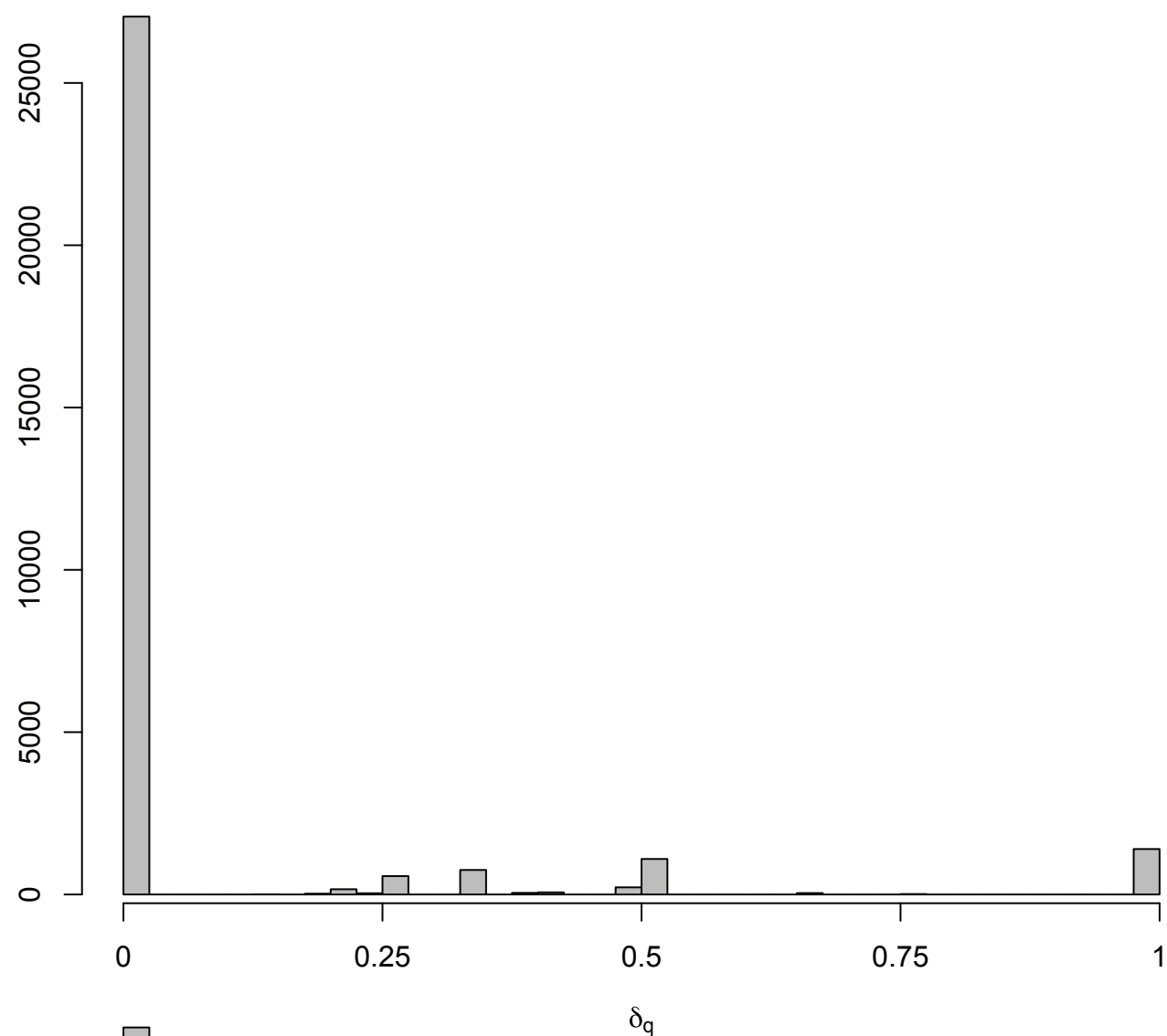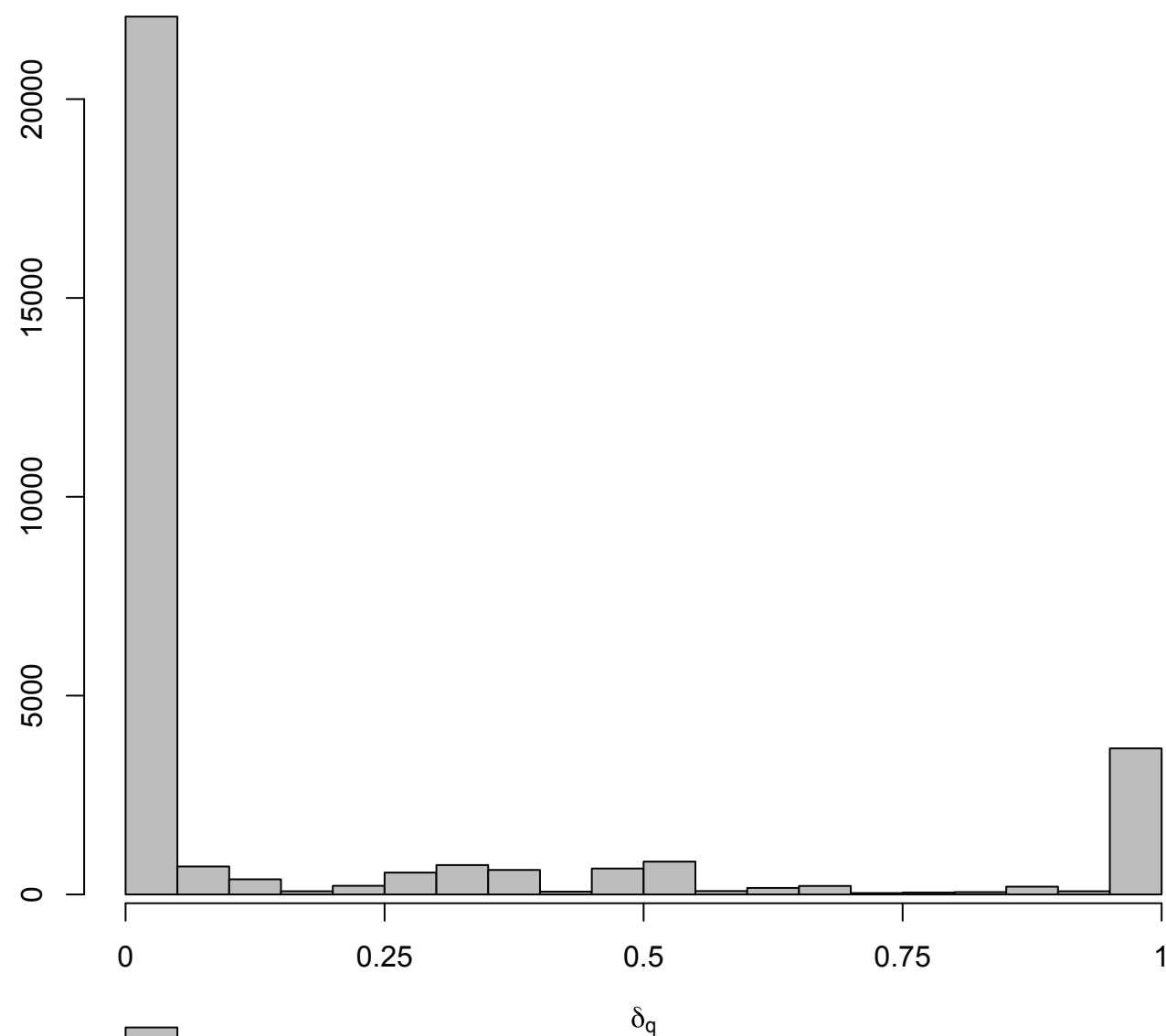

**Cyt-b**

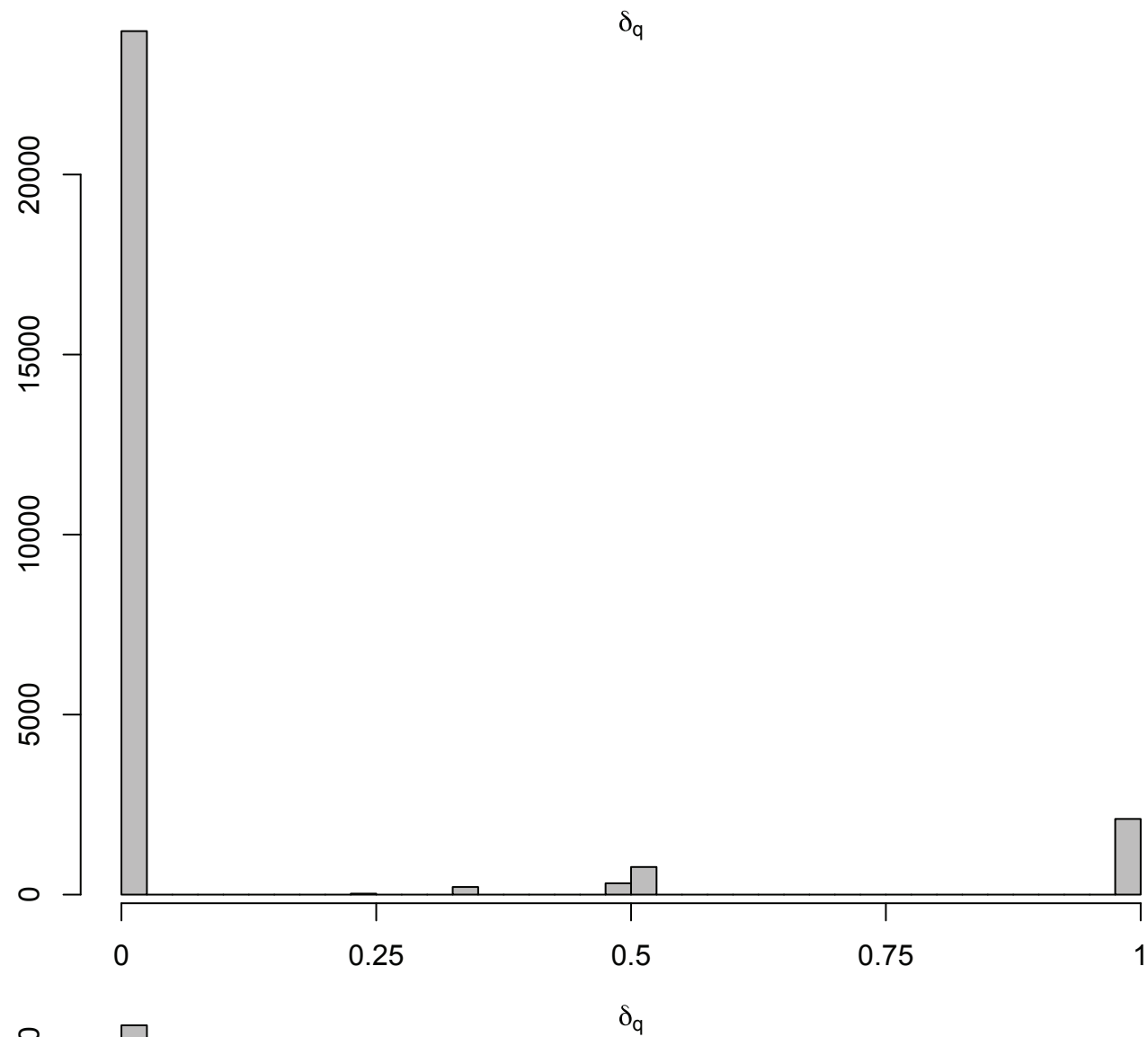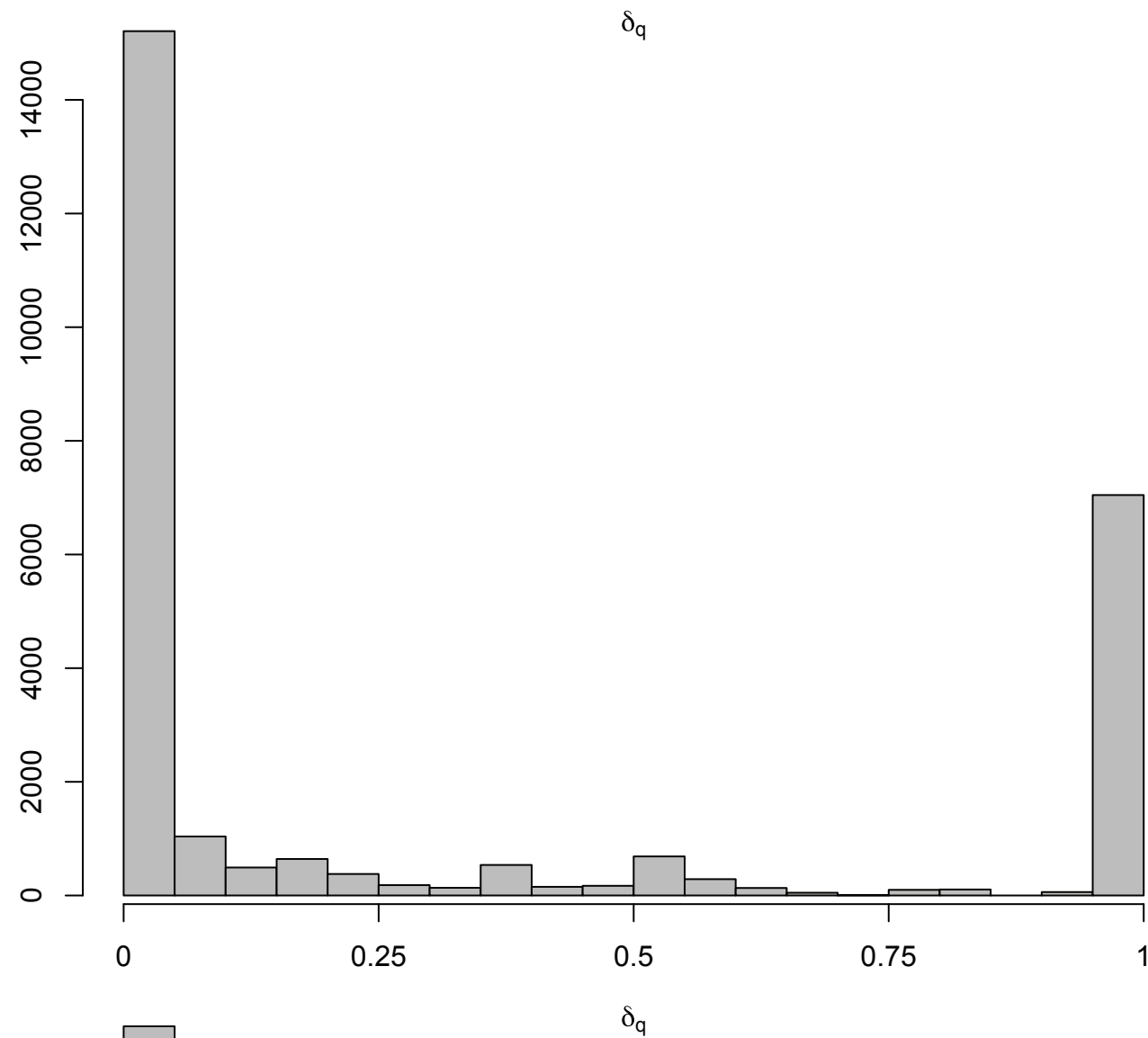

**COI**

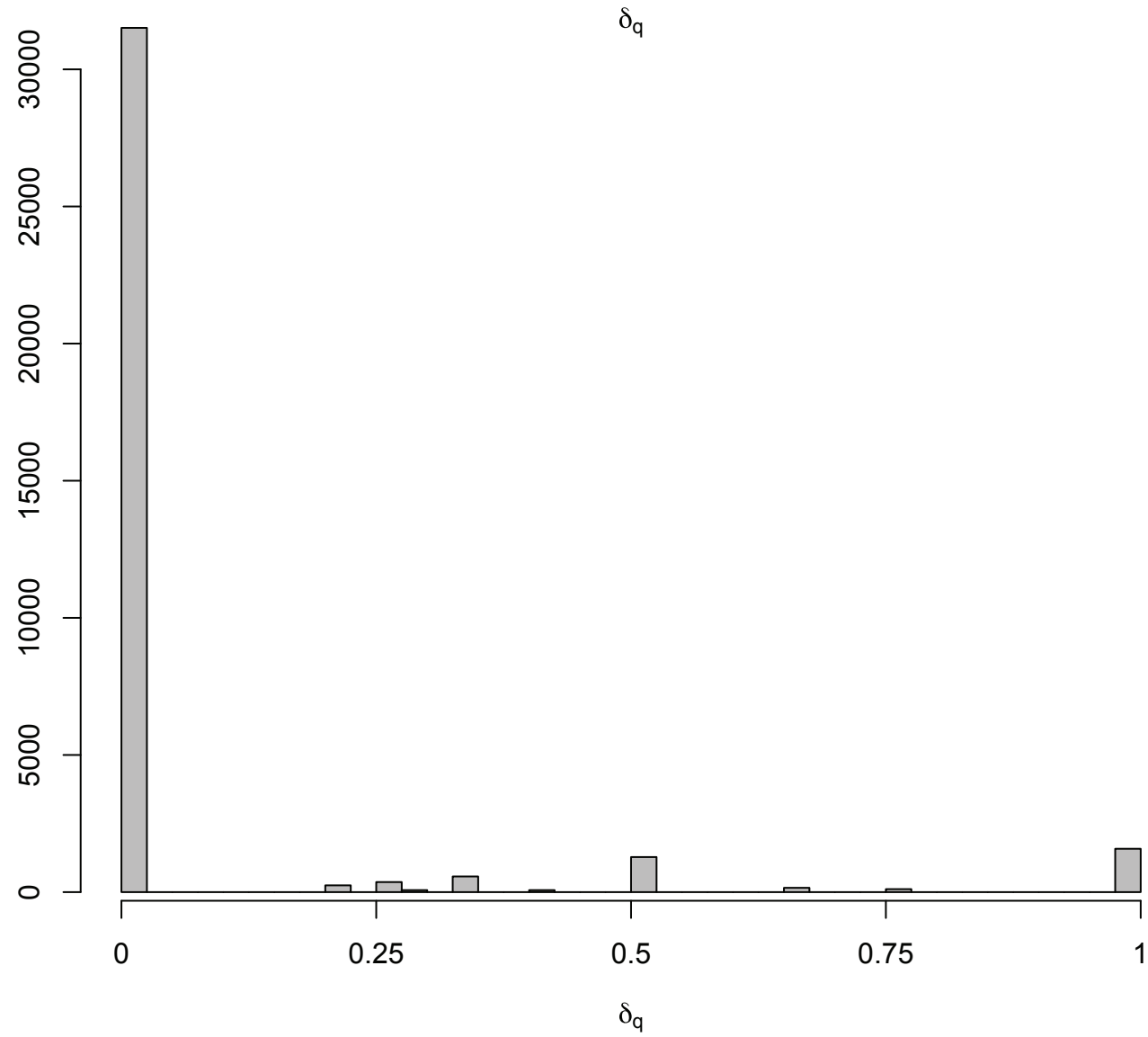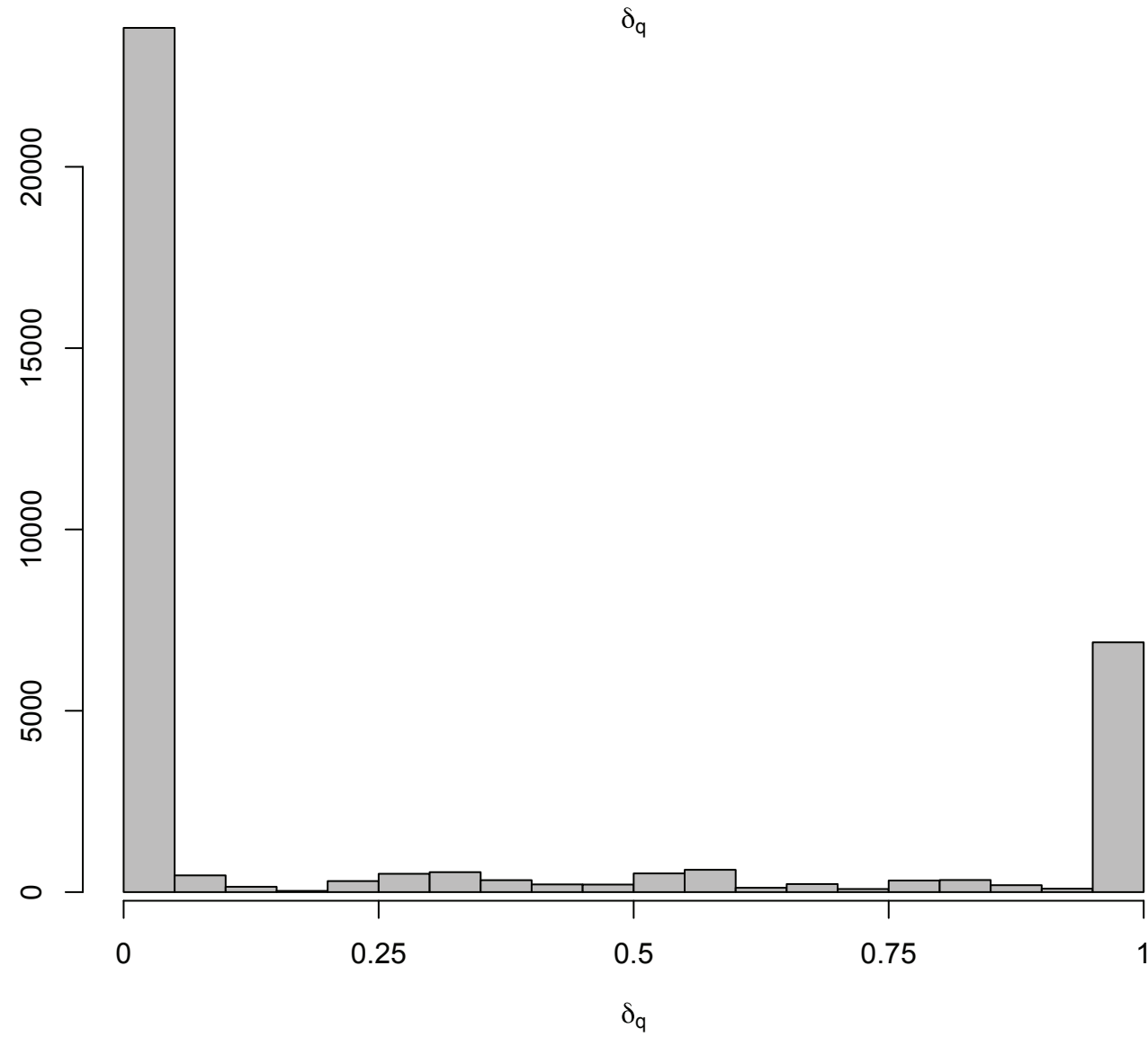

Supplement: Supplementary file 2 — Figure S2. Pairwise distances (uncorrected p‐distances) or Tamura Nei (1993) (TN93) corrected genetic distances were calculated from the hadal liparid single loci datasets using the dna.dist function in the R.cran (ver. 4.2.1, cite) package Ape (ver.5.6‐2, cite) with either “raw” or “TN93” selected as the evolutionary model. [file ECE3-15-e71779-s004.pdf]

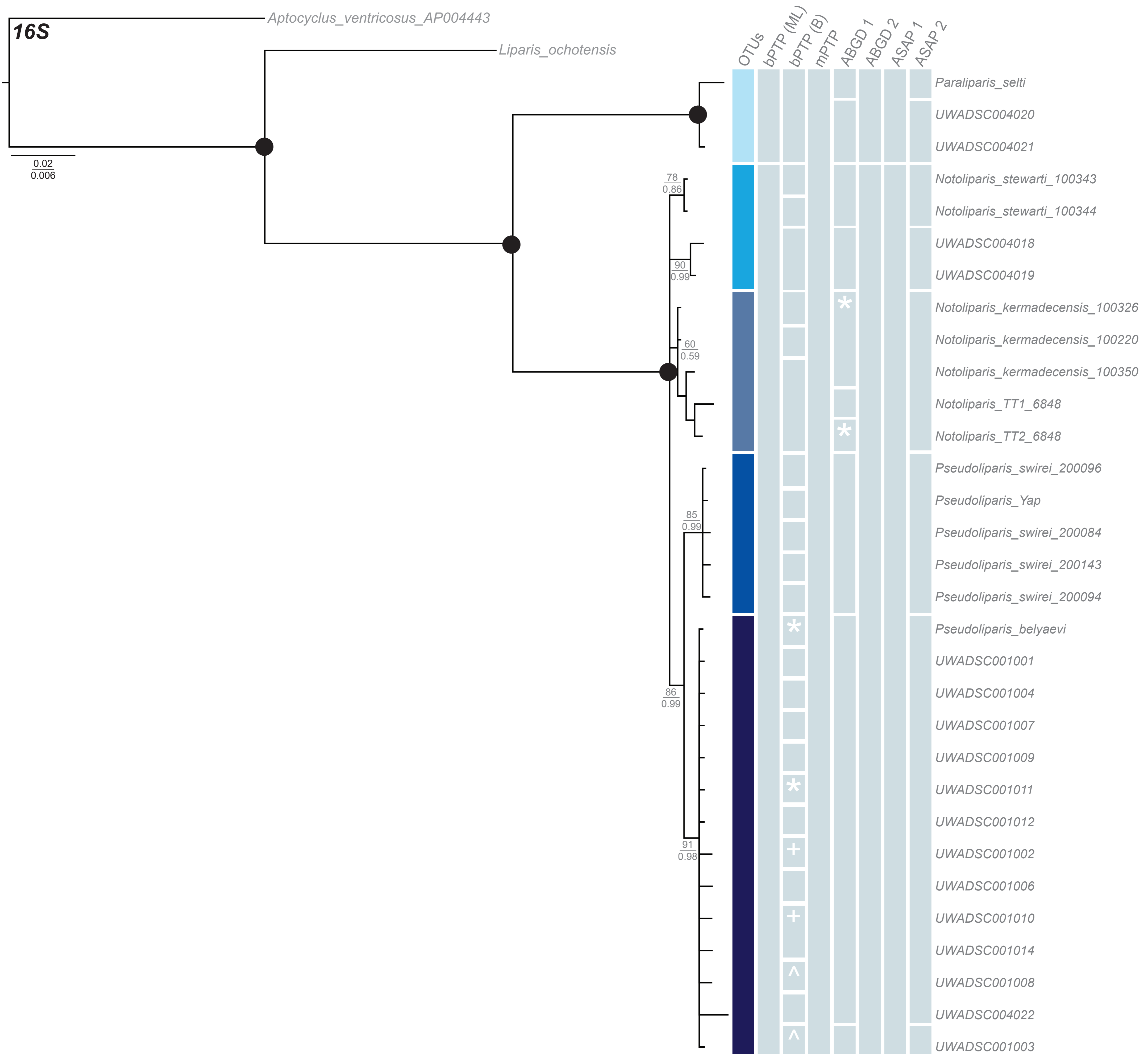

Supplement: Supplementary file 3 — Figure S3. A maximum likelihood and Bayesian phylogeny of Paraliparis c.f. selti, Pseudoliparis and Notoliparis using the mtDNA gene region, 16S. The tree is rooted with Aptocyclus ventricosus and an additional outgroup, Liparis ochotensis is shown. Nodes with ultrafast bootstrap (UF) support values of 95 or higher and Bayesian posterior probability (PP) support values of 0.95 or higher are denoted by a black circular node shape. All other support values are retained with the ML UF support values presented above and the Bayesian PP support values below the fraction midline. Boxes represent the operational taxonomic units (OTUs) followed by the partition results for all species delimitation analyses (bPTP, mPTP, ABGD and ASAP). Shapes presented within the boxes under species delimitation results represent where a delimitation result has clustered samples, but these samples are not directly next to each other within the phylogenetic tree. [file ECE3-15-e71779-s007.pdf]

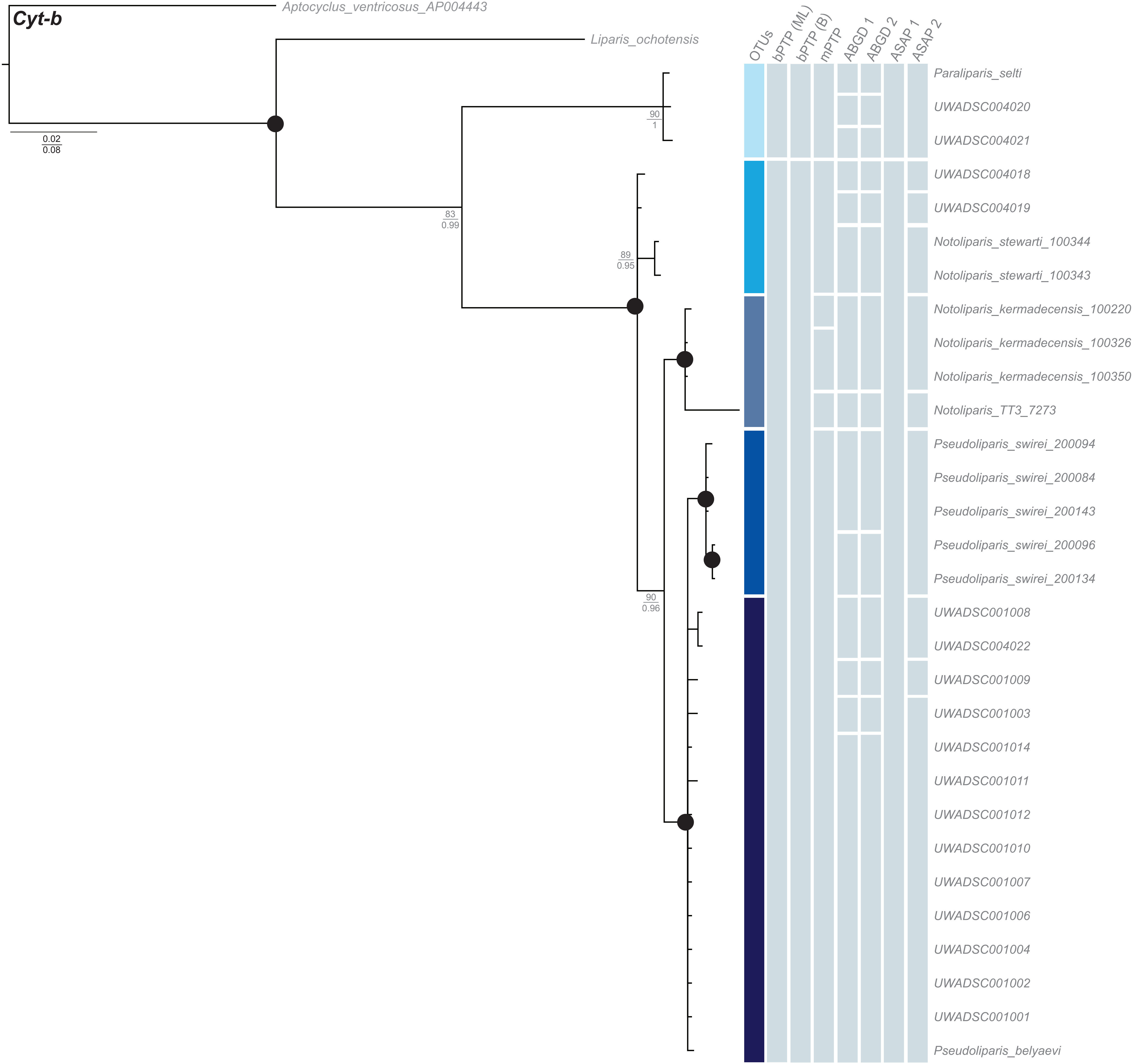

Supplement: Supplementary file 4 — Figure S4. A maximum likelihood and Bayesian phylogeny of Paraliparis c.f. selti, Pseudoliparis and Notoliparis using the mtDNA gene region, Cyt‐b. The tree is rooted with Aptocyclus ventricosus and an additional outgroup, Liparis ochotensis is shown. Nodes with ultrafast bootstrap (UF) support values of 95 or higher and Bayesian posterior probability (PP) support values of 0.95 or higher are denoted by a black circular node shape. All other support values are retained with the ML UF support values presented above and the Bayesian PP support values below the fraction midline. Boxes represent the operational taxonomic units (OTUs) followed by the partition results for all species delimitation analyses (bPTP, mPTP, ABGD and ASAP). [file ECE3-15-e71779-s006.pdf]

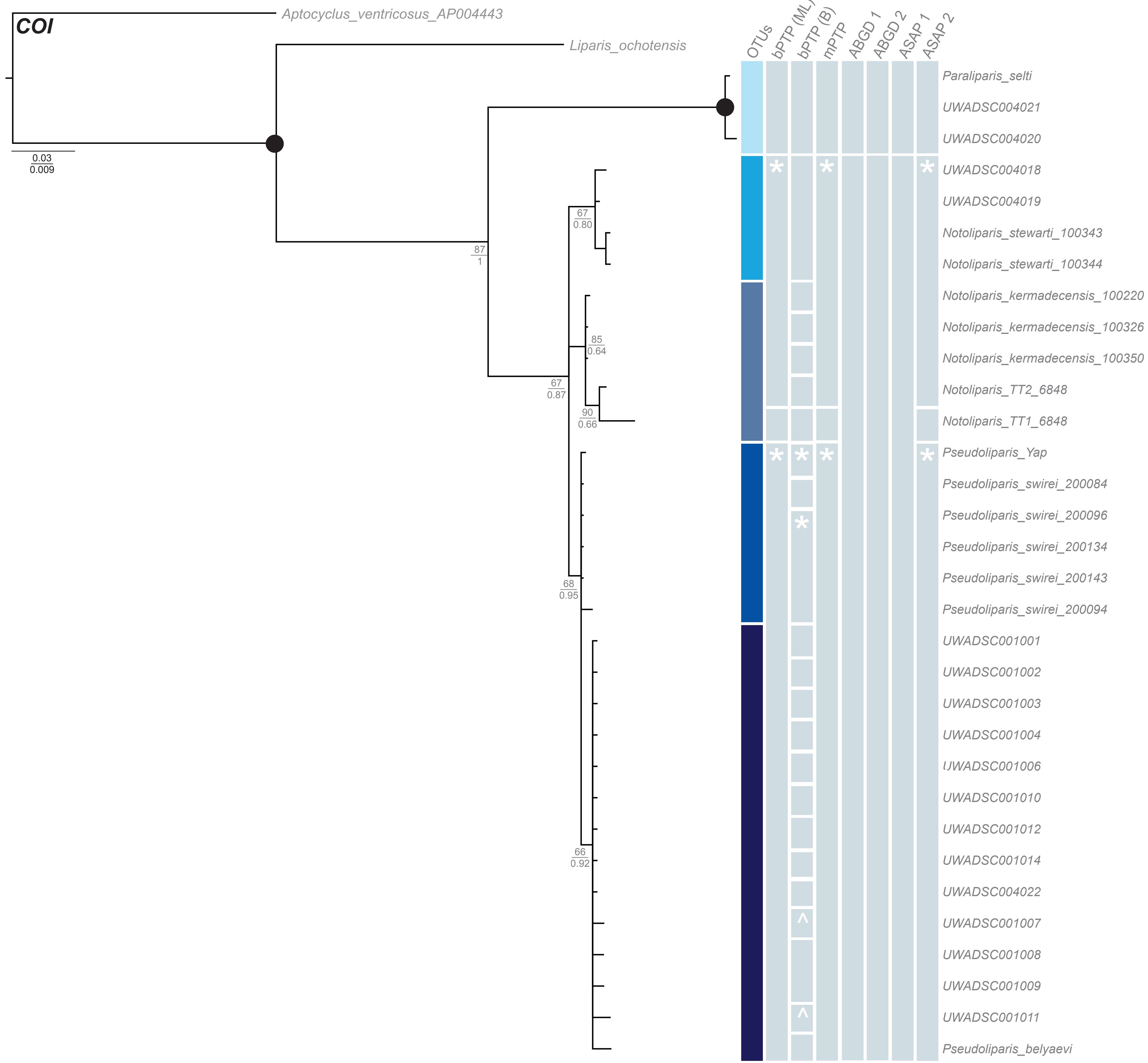

Supplement: Supplementary file 5 — Figure S5. A maximum likelihood and Bayesian phylogeny of Paraliparis c.f. selti, Pseudoliparis and Notoliparis using the mtDNA gene region, COI. The tree is rooted with Aptocyclus ventricosus and an additional outgroup, Liparis ochotensis is shown. Nodes with ultrafast bootstrap (UF) support values of 95 or higher and Bayesian posterior probability (PP) support values of 0.95 or higher are denoted by a black circular node shape. All other support values are retained with the ML UF support values presented above and the Bayesian PP support values below the fraction midline. Boxes represent the operational taxonomic units (OTUs) followed by the partition results for all species delimitation analyses (bPTP, mPTP, ABGD and ASAP). Shapes presented within the boxes under species delimitation results represent where a delimitation result has clustered samples, but these samples are not directly next to each other within the phylogenetic tree. [file ECE3-15-e71779-s002.pdf]

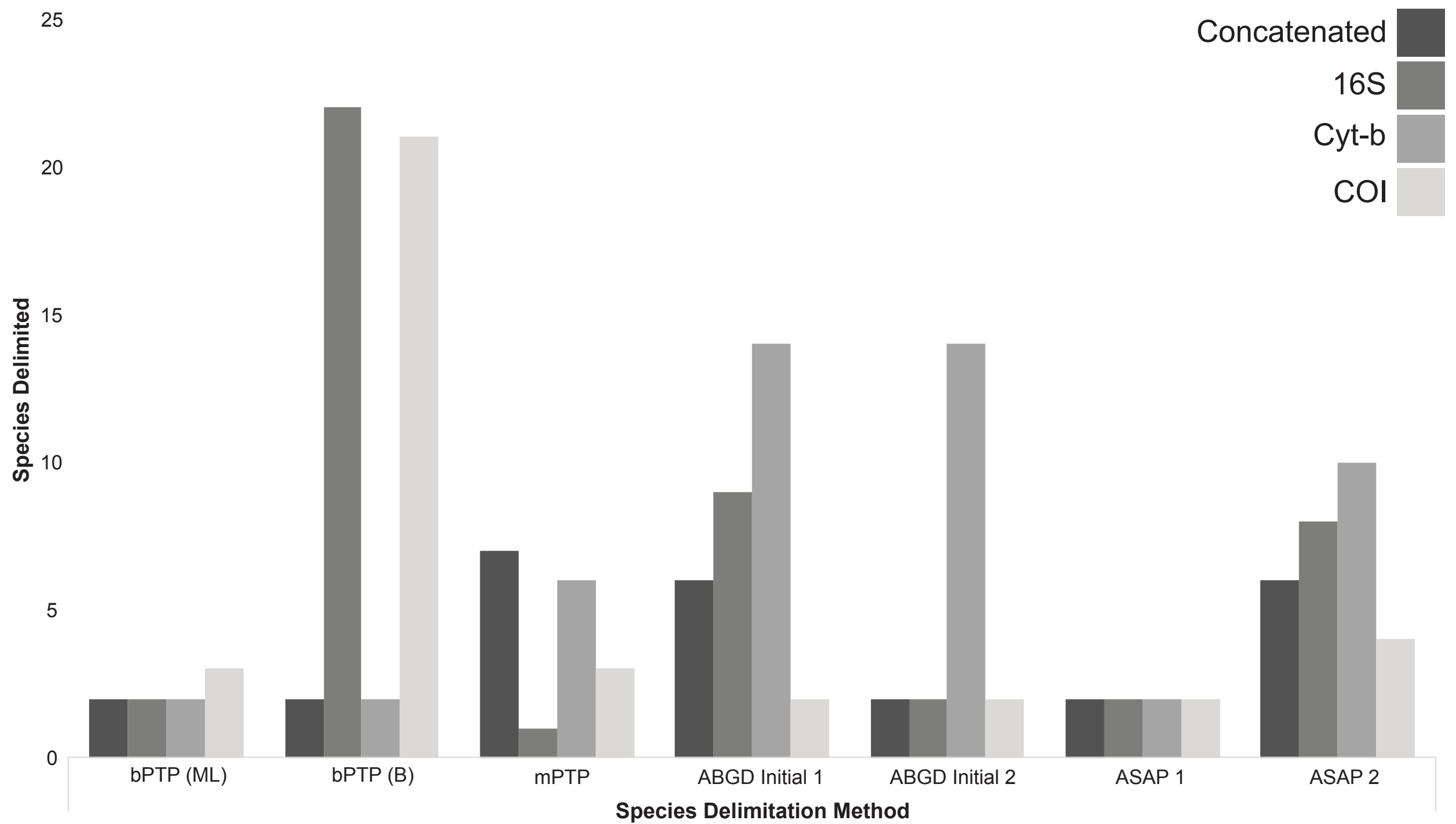

Supplement: Supplementary file 6 — Figure S6. A comparison of the species delimitation results determined per genetic dataset. For the concatenated dataset, bPTP recovered two taxonomic units, mPTP yielded 7 species‐level groups, species delimitation based on the “TN93” corrected‐genetic distances using ABGD detected between six (initial partition 1, p = 0.001) and two (initial partition 2, p = 0.00167) units using the K80, TS/TV = 2.0 model and ASAP delimited between two (ASAP‐score = 1.00) and seven (ASAP‐score = 2.00) species, also using the K80, TS/TV = 2.0 model. For 16S, bPTP recovered between two (maximum likelihood solution) and 21 (highest Bayesian support solution) taxonomic units, mPTP yielded one species‐level group, species delimitation based on the “TN93” corrected‐genetic distances using ABGD detected between nine (initial partition 1, p = 0.001) and two (initial partition 2, p = 0.00167) units using the K80, TS/TV = 2.0 model and ASAP delimited between two (ASAP‐score = 1.00) and eight (ASAP‐score = 3.00) species, also using the K80, TS/TV = 2.0 model. For Cyt‐b, bPTP yielded two taxonomic units, mPTP delimited four species overall, ABGD detected between 14 (initial partition 1, p = 0.001, initial partition 2, p = 0.00167) species units using the K80, TS/TV = 2.0 and ASAP recovered between 2 (ASAP‐score = 1.00) and 10 (ASAP‐score = 2.00) species, also using the K80, TS/TV = 2.0 model and based on the “TN93” corrected‐genetic distances. For COI, bPTP recovered between three (maximum likelihood solution) and 21 taxonomic units (highest Bayesian support solution), mPTP delimited 3 species overall, for ABGDs initial partition 1 and 2, the program detected 2 species units (initial partition 1, p = 0.001, initial partition 2, p = 0.00167) using the K80, TS/TV = 2.0 and ASAP recovered between 2 (ASAP‐score = 1.00) and 4 (ASAP‐score = 2.50) species, also using the “TN93” corrected‐genetic distances and the K80, TS/TV = 2.0 model. [file ECE3-15-e71779-s001.pdf]
